# Pedestrian and Vehicle Detection in Autonomous Vehicle Perception Systems—A Review

**DOI:** 10.3390/s21217267

**Published:** 2021-10-31

**Authors:** Luiz G. Galvao, Maysam Abbod, Tatiana Kalganova, Vasile Palade, Md Nazmul Huda

**Affiliations:** 1 Department of Electronic and Electrical Engineering, Brunel University London, Kingston Ln, Uxbridge UB8 3PH, UK; maysam.abbod@brunel.ac.uk (M.A.); tatiana.kalganova@brunel.ac.uk (T.K.); 2 Centre for Data Science, Coventry University, Priory Road, Coventry CV1 5FB, UK; vasile.palade@coventry.ac.uk

**Keywords:** autonomous vehicle, vehicle detection, pedestrian detection, generic object detection, deep learning, traditional technique

## Abstract

Autonomous Vehicles (AVs) have the potential to solve many traffic problems, such as accidents, congestion and pollution. However, there are still challenges to overcome, for instance, AVs need to accurately perceive their environment to safely navigate in busy urban scenarios. The aim of this paper is to review recent articles on computer vision techniques that can be used to build an AV perception system. AV perception systems need to accurately detect non-static objects and predict their behaviour, as well as to detect static objects and recognise the information they are providing. This paper, in particular, focuses on the computer vision techniques used to detect pedestrians and vehicles. There have been many papers and reviews on pedestrians and vehicles detection so far. However, most of the past papers only reviewed pedestrian or vehicle detection separately. This review aims to present an overview of the AV systems in general, and then review and investigate several detection computer vision techniques for pedestrians and vehicles. The review concludes that both traditional and Deep Learning (DL) techniques have been used for pedestrian and vehicle detection; however, DL techniques have shown the best results. Although good detection results have been achieved for pedestrians and vehicles, the current algorithms still struggle to detect small, occluded, and truncated objects. In addition, there is limited research on how to improve detection performance in difficult light and weather conditions. Most of the algorithms have been tested on well-recognised datasets such as Caltech and KITTI; however, these datasets have their own limitations. Therefore, this paper recommends that future works should be implemented on more new challenging datasets, such as PIE and BDD100K.

## 1. Introduction

In recent years, many countries around the world have been facing road traffic issues such as accidents, congestion, and pollution. According to WHO [1], in 2016, the number of fatalities due to road traffic accidents reached 1.35 million, and approximately 20 to 50 million people are injured each year. In addition, it was reported that road traffic accidents are the primary reason for the deaths of children and young adults. Human error and imprudence, for instance, fatigue, drink-and-driving, using mobile phones while driving and speeding, are two of the main factors that contribute to these extreme numbers [2]. In order to decrease road traffic accidents and fatalities, the following measures were presented: enforce legislation to avoid human error and imprudence, improve vehicle safety to avoid or mitigate collisions, and post-crash care to increase the chance of saving lives. The advanced driver assistance system (ADAS) is one of the proposed solutions to make vehicles safer and to reduce driver error. According to IIHS-HLDI [3], several car manufacturers have adopted ADAS technology and this has already reduced traffic accidents. However, the technology has its limitations because it still depends on the driver’s actions and road users’ acceptance [3]. Strategies have also been proposed to reduce congestion and pollution, for example, making road improvements and using other methods of transportation (e.g., cycling, trains, buses, etc.); however, it is expected that by 2050 the urban population will double [4] and, in the next twelve years, the number of cars on the road will be approximately two billion [5]. Although some solutions were proposed to deal with the mentioned traffic issues, they have their limitations and might not be sufficient. A promising solution that has been highly investigated in the past decades is AV. However, many challenges need to be solved to make AV available on public roads. For instance, AV needs to perceive their environments to safely navigate in busy urban scenarios to prevent collisions. Hence, this work reviews and investigate the computer vision techniques that can be used to create a perception system for AV. There are two main approaches to develop a perception system for an AV, pure-vision based or sensor–fusion based. This paper only reviews a pure-vision based system, where a monocular camera is mounted on the vehicle’s dashboard. An AV perception system must detect static and non-static objects, recognise the information provided by the static objects, and predict the non-static objects’ behaviour. Due to the space limitation, the review will be split into three parts. This article, which is the first, will review the computer vision techniques that have been used to detect pedestrians and vehicles. The subsequent review papers will review the computer vision techniques used to detect and recognise traffic signs and traffic lights, and the technique used to predict the non-static objects behaviour.

Benenson et al. [6], Nguyen et al. [7], Antonio and Romero [8], Ragesh and Rajesh [9], Gilroy et al. [10] performed a review on pedestrian detection algorithms. At the time, Benenson et al. [6] had reviewed the most relevant algorithms for the 10 previous years. They reported that the main ways to improve detection performance were to acquire improved features, use the Deformable Part Model (DPM), use decision forest and DL. Although they reviewed DL techniques, only a limited amount of work was covered, since DL was starting to emerge at the time. In addition, the authors only focused on the works that were mainly trained and tested using the Caltech dataset. Nguyen et al. [7] explored the developments and challenges of pedestrian detection algorithms. They covered the state-of-the-art algorithms between 2010 and 2015, and most of them were based on traditional techniques. They concluded that pedestrian detection algorithms’ performance are mostly dependent on the extracted features, which are used to build the descriptor. The authors only focused on the algorithms that were trained and tested on the Caltech dataset. Antonio and Romero [8] reported mainly DL algorithms, but only a few of them were reported and they did not provide sufficient and clear details of the algorithms, such as the methods used, the problem that they were handling, the datasets used, and the results acquired. Ragesh and Rajesh [9] present an extensive review, covering the specific requirements for ADAS systems; they covered the traditional and DL techniques used to detect pedestrians, the different metrics to evaluate pedestrian detection algorithms, the trends and suggestions for further work. However, the mentioned DL algorithms were limited, for instance, RNN (LSTM), encoder–decoder architectures and ensembles were not mentioned. They also only reported algorithms that were trained and tested on Caltech and CityScape datasets. Gilroy et al. [10] is one of the most recent pedestrian detection review papers, but their review is only focused on the algorithms used to handle occluded objects.

Sivaraman and Trivedi [11], Mukhtar et al. [12], Abdulrahim and Salam [13], Antony and Suchetha [14], Shobha and Deepu [15], Abbas et al. [16] performed reviews on vehicle detection algorithms; however, they only explored traditional techniques. There are limited papers that reviews DL vehicle detection algorithms, for example, Manana et al. [17] reviewed vehicle detection system that uses DL technique; however, they covered systems that use satellites images and images that are acquired from a mono camera mounted at the back of the vehicle. Manana et al. [17], Wang et al. [18], Meng et al. [19] only referred DL techniques that are related to general object detection, and not in the on-road vehicle detection domain. Kiran et al. [20], Yang and Pun-Cheng [21] mentioned DL techniques for on-road vehicle detection but only a few works were mentioned. Arnold et al. [22] only reviewed 3D vehicle detection techniques. Haris and Glowacz [23] only compared the performance of the main general object detection algorithms to detect road objects. Different from the previous works, this paper:

- Reviews not only pedestrian or vehicle detection algorithms, but both of them. Since many pedestrian and vehicle detection algorithms used a modified version or techniques of generic object detection algorithms, these are also reviewed;
- Briefly presents the most important traditional techniques and focuses more on the DL techniques for generic objects, pedestrian, and vehicles detection algorithms;
- Reports works from 2012 until 2021 and works that were performed on different datasets, such as Caltech, KITTI, BDD100K and others;
- Summarises the main information acquired from the reviewed pedestrian and vehicle detection works in tables. The tables report the methods, the problem that the algorithms are trying to solve, the datasets used and the results acquired.

This paper is structured as follows: Section 2 introduces AV system discussing its history, benefits, challenges, taxonomy, and system architecture; Section 3 presents the evolution of the most relevant traditional and DL techniques used to detect generic objects; Section 4 reviews traditional and DL techniques used to detect vehicles; Section 5 reviews traditional and DL techniques used to detect pedestrians; and Section 6 discusses the main findings and if the reviewed algorithms can be used in an AV perception systems.

## 2. Autonomous Vehicle Systems

The idea of AV started around 1920 and, at the time, they were called “phantom auto” since the vehicle did not have a driver and it was remote-controlled [24]. AVs only progressed in the 1980s when Pomerleau [25] created the project “Autonomous Land Vehicle In a Neural Network”, where they concluded that neural networks could make a big contribution to autonomous navigation systems. The Defence Advanced Research Projects Agency (DARPA) has organised the first Grand challenge in 2004, where the objective was to motivate research and development of AVs. DARPA organised other grand challenge events in 2005 and 2007 also known as the Urban Challenge [26]. In 2008, Rio Tinto started the trials of autonomous haul truck fleet to transport ore and waste material in Pilbara. Nowadays, they have more than 130 autonomous trucks [27]. In 2009 Google secretly started developing its first AV and they were able to pass the first self-driving test on 1 May 2012 in Las Vegas [28]. The UK government launched a driver-less competition in 2014 to support and encourage AV [29]. Between 2010 and 2017 major automotive manufacturers such as General Motor, BMW, Nissan, Volkswagen, Tesla, Volvo, Mercedes-Benz, Toyota and Audi recognised the potential benefits of AVs; therefore, they adopted the concept and started their research and development [30,31]. In 2019, the European Parliament and Council released the Regulation (EU) 2019/2144 for the first time specifying requirements associated with automated and fully automated vehicles [32]. A big step for AV was achieved when Waymo (Google self-driving wing became Waymo in 2016) reported that their “Waymo Driver” reached 20 million self-driven miles and 15 billion simulated miles [33]. This is an important achievement since these self-driven miles are considerable training experience that can be used as the dataset for other AI systems.

### 2.1. Benefits and Challenges

AVs are expected to offer many benefits, for example, to follow the traffic law and to have a quick response to unexpected scenarios; therefore, a significant reduction in road traffic accidents is expected since most of them are caused by human error and imprudence. AV is expected to foresee the behaviour of the vehicle ahead, by doing so, it can reduce braking, acceleration and consequently reduce fuel consumption, air pollution, traffic shock-wave propagation and congestion [34]. Yet, it faces many challenges that need to be tackled; for instance, AV would replace taxis, trucks and buses drivers, as a consequence, the number of unemployed workers would increase. Table 1 presents several more benefits and challenges of AV systems.

### 2.2. AV Taxonomy

Society of Automotive Engineers (SAE) created the J3016-2018 guidelines outlining the taxonomy and definitions for driving automation systems. The document describes the six levels of driving automation: in Level 0 there is no automation; in Level 1 there is some automation assistance, such as ADAS features that can control the steering or speed. However, the driver is responsible to supervise and act when required; Level 2 enables partial driving automation where the autonomous system can control both steering and speed; however, the driver is still responsible to observe the environment and support the autonomous system; Level 3 enables conditional driving automation where the car is fully automated when certain conditions are met, for instance, good weather and visibility. When conditions are not favourable, the driver must be in control; Level 4 enables high automation where the automated system does not require the driver to be in control; however, the system only works if certain conditions are met; and Level 5 enables full driving automation where the automated system is always under control and can drive in any condition [36,37].

### 2.3. AV System Architecture

**Figure 1:**
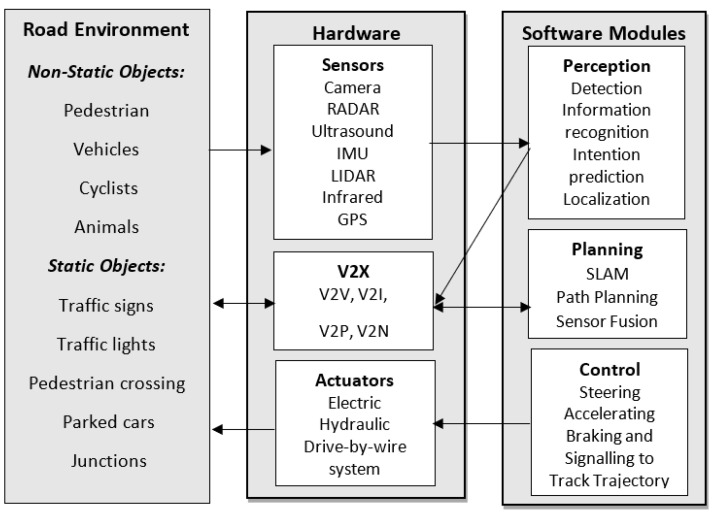
AV architecture: hardware requirements are sensors, V2X communication device and actuators. Software modules are perception, planning and control.

AV functional requirement can be compared to an autonomous mobile robot system, it requires Perception, Communication, Localisation, Path Planning and Trajectory, and Motion Control [38,39]. These functional requirements in AV systems are commonly referred to as sense, plan, and act, or perception, planning and control [40]. The functional requirements should answer the following question: “Where the AV is ?”, “What is around the AV ?”, “What will happen next ?” and “What should the AV do ?” [33]. An AV block diagram proposed by [40] is depicted in Figure 1.

#### 2.3.1. Perception

Perception is an important topic, since it may enable the AV to perform reliable, efficient, and safe driving [31]. It should answer the “Where the AV is?” and “What is around the AV?” questions. The perception module uses the raw data information from the environment, acquired by sensors and communication devices, to extract road features, detect road objects and predict their behaviour. Furthermore, the raw data is used to enable the AV to execute the Simultaneous Localisation and Mapping (SLAM) task [36]. For a detailed AV literature review, please refer to [41].

Passive (receive and measure existing energy) and/or active sensors (measures reflected signals that have been transmitted by it) can be used to perceive the environment. Passive sensors generally used are Charge-Coupled Devices (CDD) or Complementary metal-oxide semiconductors (CMOS) cameras. Active sensors generally used are Light Detection and Ranging (LIDAR), long/medium/short Radio Detection and Ranging (RADAR), ultrasound/Sound Navigation System (SONAR), Inertial Measurement Unit (IMU) and Global Navigation Satellite Systems (GNSS). The advantages and disadvantages for each sensor are described in Table 2. It is observed that each sensor has their strengths and weaknesses, for example, radar sensors work well in the dark, it is not affected by extreme weather and can accurately detect speed, but it has low resolution. An approach to overcome the deficiency of each sensor is to use sensor fusion technology, where data from multiple sensors are combined to attain enhanced information. Recently, AV systems have been implemented using two main methods, a pure vision-based approach where only cameras and computer vision techniques are used, and a sensor fusion approach where information from multiple sensors and computer vision techniques are used (e.g., cameras, LIDAR, RADAR, etc.) [42]. For example, Tesla uses a pure vision-based technique to acquire information from the traffic scene, whereas, Waymo uses computer vision and fusion of advanced sensors. The main advantages of a pure vision-based system are that, many new cars already have cameras and once an AI computer vision system is created, it can be easily deployed, cameras are much cheaper than LIDAR, cameras have more resolution, and LIDAR systems need to pre-map the environment first, whereas, in the pure vision-based system everything happens at once. For these reasons the remaining sections of this paper mainly focus on a pure vision-based system.

#### 2.3.2. Planning

Once the AV can perceive its environment, the next stage is to plan the AV actions to achieve its goal. It should answer “What will happen next ?” and “What should the AV do?” questions. The planning stage is generally subdivided into three tasks, mission, behaviour, and motion planner [40]. The mission planner is responsible to assign a goal (e.g., pickup/drop-off task) to the AV and choose the best routine to complete the assigned goal. The behaviour planner takes into consideration the interaction between other traffic agents, as well as the available traffic rules to decide what behaviour the AV should perform, for example, should the AV change lane, stop, turn left or right. Finally, the motion planner is responsible to generate paths to perform the behaviour determined by the behaviour planner without collision. The planning stage has been implemented using traditional techniques such as the Voronoi diagram, occupancy grid algorithm, or driving corridors diagram. However, these approaches are not suitable for complex urban scenarios where the interaction between different traffic agents and the different traffic rules need to be taken into account. Lately, many researchers have been using machine learning (ML) such as CNN, Deep Reinforcement Learning, or hybrid systems where ML and traditional techniques are jointly used [36].

#### 2.3.3. Act

The information acquired from the planning stage, is used by the control stage to perform the actual movements of the AV, which are performed by sending steering, acceleration, braking, and signalling commands to the actuators. The most appropriate and advanced way to transfers the commands to actuators are the Drive-by-Wire system. The control system is responsible to generate and track trajectories, as well as to use controllers to make sure that the desired trajectories are performed. Trajectories generation are usually achieved either by sensor-based or dynamics based. Sensor-based approaches are more suitable for robotics while dynamic based are suitable for vehicles. The most used methods to track trajectories are geometric or model-based. A feedback controller such as Proportional-Integral-Derivative (PID) is usually used to make sure that the AV is not deviating from the target trajectories. However, feedback controllers have their limitations, for example, the system will only respond to errors when they occur [40]. Two degrees of freedom controller, which is a combination of feedback and feedforward controllers, have been proposed to overcome the limitations of the feedback controller. In this type of controller, a model reference of the system is also used, which help the system to predict the AV motion with more details.

## 3. General Object Detection

AVs should perceive static objects such as parked cars, road works, road signs, traffic lights, and so forth; as well as non-static objects such as pedestrians, animals, cyclists, large/medium/small vehicles, motorcyclists, and so forth. Of all these road objects this work concentrates on pedestrians and vehicles, since the former are the most vulnerable ones and the latter are the ones that most interacts with the ego vehicle. Several generic object detection algorithms have been modified to detect pedestrians or vehicles, and for this reason, this section review the most relevant generic object detection algorithms and the next sections review vehicles and pedestrians detection algorithms. If the reader is already familiar with generic object detection algorithms please go to Section 4 or Section 5.

Human eyes can easily extract features of an image/video to perceive and interpret a scene. This capability has been evolving over millions of years [43]. Computer vision scientists and engineers have worked on many computer vision tasks to enable computers to achieve these capabilities [44], for example:

- Image classification (Recognition): extracts features and information from an image to predict in which category it belongs to.
- Object detection: detects single or multiple objects in an image, surrounds each one of them with a bounding box and identify their locations.
- Object tracking: predicts the objects motions.
- Object segmentation: once each object is detected in an image, a pixel-wise mask for each object is created instead of separating them with surrounding boxes.

In the literature, computer vision, object recognition and detection algorithms have been classified as Traditional or Deep Learning (DL) techniques [45].

### 3.1. Traditional Techniques

The most known traditional techniques for feature extraction are Scale Invariant Feature Transform (SIFT) [46], Viola-Jones rectangles, Haar-Like-Wavelets, Histogram of Oriented Gradient (HOG) [47], Edge-Orientation-Histograms (EOH), Optical Flow (motion), Implicit Shape Model (ISM), SHAPELET, Self-similarity Shannels (SSC), Speeded Up Robust Features (SURF), Maximally Stable Extremal Regions (MSER), Integral Channels Features (ICF) and Aggregated Channel Features (ACF). The most known traditional techniques for object classification are linear Support-Vector-Machines (SVM), Graphical Model, non-linear SVM, Adaptive Boosting (AdaBoost), Artificial Neural Networks (ANN), and MPL-Boost. From the listed classifiers, linear SVM is the most used since it requires less memory, it is fast to train and classify. Table 3 presents the advantages and disadvantages of the other learning algorithms.

The SIFT algorithm presented by Lowe [46,48] was a remarkable work since the algorithm was able to find features that are invariant to scale, and rotation to create an object recognition system that is robust to partial occlusion, cluttering, noise and change in illumination. The algorithm is composed of many stages, the first step is called “scale-space extrema detection”, which uses a difference-of-Gaussian function to detect potential key points in the image that are invariant to scale and orientation. In the second step “keypoint localisation”, the location and scale of each one of the key points are determined and only the stable ones are selected. In the third step, one or more orientations are assigned for each key point. Regions around the key points are sampled to create an orientation histogram that quantises the direction of the key points in 36 bins ranging from 0${}^{\circ }$ to 360${}^{\circ }$. The gradient magnitude of the key points is used to vote on the 36 bins, only the highest peak and the peaks that have 80% of the highest peak value is selected as the key point orientation. By the end of the third step, each key point should have a location, scale, and orientation. The fourth step uses the magnitude and direction from the previous steps to create a key descriptor vector. Lastly, the key descriptor vector is used to recognise objects by matching the key points of a new image with the key points descriptor vector database. This is achieved by performing nearest neighbour indexing using Best-Bin-First (BBF) sort algorithm and by applying Hough transform (HT) to find key points clusters. The least-square method was used to verify if the new image is related to the chosen image in the database. The drawbacks of the SIFT algorithm, are that the repeatability of key points is not persistent in dynamic objects (e.g., humans), the dimension of the feature descriptor vector is considerably high in which affects the matching procedure, and the algorithm is patented.

Viola-Jones algorithm is another object recognition algorithm presented by Viola and Jones [49] where they aimed to recognise human faces in an image using Harr-like feature extractors. Their algorithm has four main aspects: firstly Haar-like feature extractor was applied in the image to select discriminative features; secondly, an Integral Image algorithm was used to simplify the image representation which enable faster computation; thirdly the overall number of features was decreased by using AdaBoost learning algorithm; and lastly, cascade classifier was used to reject background information and focus more on the regions where it is likely to have the object of interest. The algorithm has shown to be very fast since it processes a 384 × 288 pixel image in 0.067 s, using a 700 Mhz Pentium III processor. In addition, it achieved detection rates up to 91.4% with 50 false detections. Although the algorithm is fast and achieves good accuracy, its training time is slow and it is not suitable to describe general objects.

Dalal and Triggs [47] introduced the HOG algorithm with the aim to detect humans in digital images. In the HOG algorithm, the input images is processed with vertical and horizontal gradient filters to yield gradient magnitude and direction. The filtered images is first divided into 8x8 pixels cells and subsequently blocks of 2 × 2 cells with a fifty per cent overlap. Orientation histograms for each cell (descriptor cells) are created, the histograms quantise the calculated gradient direction into 9 bins ranging from 0${}^{\circ }$ to 180${}^{\circ }$ and the calculated magnitude is used as the vote for each respective bin. The orientation histograms for each cell that belongs to a particular block is concatenated to yield a HOG descriptor vector for that block. The HOG descriptor vectors for each block are normalised to take into consideration changes in illumination and contrast. The final HOG descriptor is a vector of all the normalised blocks that is fed into an SVM to classify if the input image is human or non-human. The algorithm was able to reduce false-positive results outperforming Haar Wavelets algorithms. The downsides of the HOG algorithm are that compared to SIFT, it requires more computation load due to the dense grid process and its performance is considerably affected when objects are occluded.

Bay et al. [50] presented the SURF algorithm, which has some similarities with SIFT algorithm, but the authors tried to simplify it. To detect invariant features in the image, the authors used a basic approximation of Hessian blob detector and integral images. This combination offers faster computation and good accuracy. Haar-wavelet and integral images were used for orientation assignment as well as to create the feature descriptor. The evaluation of the algorithm was achieved by calculating the Euclidian distance between the features in an input image and the features in the database. Authors reported that their algorithm has outperformed current state of art algorithms such as GLOH, SIFT and Principal Component Analyis (PCA)-SIFT, achieving an average recognition rate of 85.7%.

The main drawback of the previously cited traditional algorithms, is that they required handcrafted feature extractors to learn distinct descriptors of the objects. This requires experienced extractor engineers, it is time-consuming, and it is more suitable for specific domain systems.

### 3.2. DL Techniques

**Figure 2:**
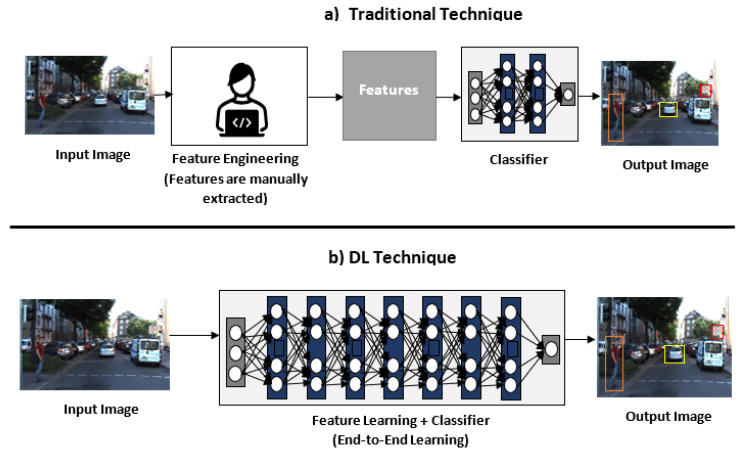
(**a**) Traditional techniques require engineers to manually extract features and then classifiers are used to learn the best descriptors. (**b**) In DL, the features are automatically extracted and learnt. (Modified version of [51].)

DL has become very popular and highly used in the past decades. According to O’Mahony et al. [51], DL has outperformed traditional computer vision algorithm in classification, segmentation, detection, and SLAM problems. The advantages of DL over traditional techniques are: it is an end-to-end approach, it is flexible because the model can be re-trained with different datasets, and it is expected to require less expertise and fine-tuning. Whereas in traditional methods, engineers must specify which features are relevant to extract, it might require fine-tuning and it is likely to be domain-specific. The main drawback of DL technique is that their performance is dependent on the network depth and the availability of the datasets. Figure 2 compares the stages for traditional computer vision and DL. This section covers the main DL techniques for general object classification and detection.

**Figure 3:**
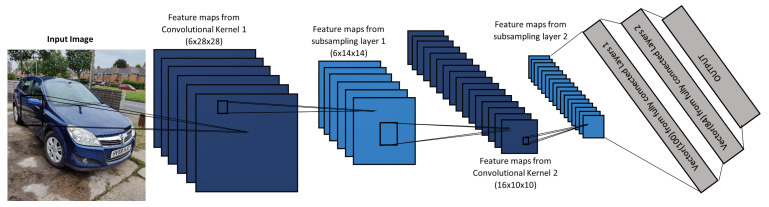
LeNet has seven layers: three convolutional layers, two sub-sampling, and two fully connected layers [52].

A seven-layer Convolutional Neural Network (CNN) system, as depicted in Figure 3, was implemented by LeCun et al. [52] to learn to extract important features from 32 × 32 × 1 handwritten characters images. The system was called LeNet and an important aspect of it, was that instead of applying the conventional gradient-based learning algorithm to learn patterns, that is limited to a linear system, they used the back-propagation algorithm. Their work was remarkable because they showed that is not required to manually extract relevant features from an input image, instead the features were automatically extracted by using CNN. In addition, they have outperformed all other character recognition techniques at the time. However, their CNN architecture is shallow, which inhibits the system to extract sufficient features to improve the algorithm generalisation and accuracy. Some authors presented a modified version of the LeNet network, for example, Lin et al. [53] used smaller convolutional kernels (3 × 3) to increase the number of extracted features and reduced the fully connected layer from 10 units to 2 units. Xie et al. [54] further improved LeNet by adding activation layers, batch normalisation layers, and online hard example mining. Li et al. [55] increased the number of convolutions kernels at some of the layers, adopted the ReLu activation function, used max-pooling instead of mean-pooling layers, and used SVM at the output layer.

**Figure 4:**
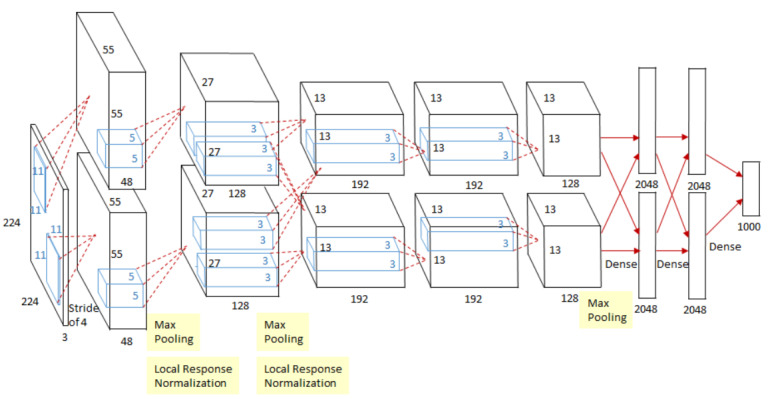
AlextNet have eleven layers: five convolutional layers and three fully connected layers that computes weights; and three max pooling layers [57].

An eight layers CNN system, similar to LeNet, called AlexNet was performed by Krizhevsky et al. [56]. At this time, the authors had access to a larger image dataset and more advanced computing resources such as powerful CPUs, GPUs, and larger memory sizes. These improvements enabled the authors to work with high-resolution colour images, to have deeper neural network architecture and to have more filters with wider dimensions. One important feature of the system was that the authors used the ReLU non-linearity activation function instead of the traditional ones, this enabled the system to train much faster and be more robust against vanishing gradient. AlexNet was tested in the ImageNet Large-Scale Visual Recognition Challenge (ILSVRC) contest which uses the ImageNet database of 1.2 million images belonging to 1000 categories. The algorithm outperformed prior state of art algorithms and it achieved error rates of 37.5% and 17.0% in the top 1 and top 5, respectively. An important conclusion that the authors reported is that, deep CNN is expected to have a better performance. Even though the ReLU activation function speeded up the training time, AlexNet still took 5 to 6 days to train 1.2 million images. This issue limited the DL engineers to optimise their algorithm, test new ideas, and construct deeper CNN. Figure 4 depicts the overall structure of the AlexNet.

After AlexNet, many other CNN work was presented as an approach for object recognition and detection problems. Although, good results have been reported on datasets such as ImageNet and PASCAL, up until 2013 no work had been done to truly understand why such good results were achieved and how activation layers of CNN systems works. Author Zeiler and Fergus [58] used the Multi-layered Deconvolutional Network technique to visualise and understand what is happening in the CNN activation layers. The process is similar to the CNN system however it does the inverse, it maps features maps to pixels space. The visualisation technique led the authors to identify AlexNet architecture limitations and to make the required change to achieve a better performance. The authors named their system as ZFNet, which has the same architecture as the AlexNet but, some of the hyper-parameters were improved. For instance, ZFNet uses a 7 × 7 filter in the first layers instead of an 11 × 11 filter and it uses a stride of 2 instead of 4. These changes allowed the system to keep more information in the first two layers, consequently improving the system performance in classifying the images. In addition, these changes reduced the chance of aliasing artefacts. ZFNet was able to reach a test error of 14.8% using the ImageNet 2012 dataset, meaning that they have outperformed AlexNet by 1.7%. However, the algorithm still requires a large number of parameters and take a considerable amount of time to train.

**Figure 5:**
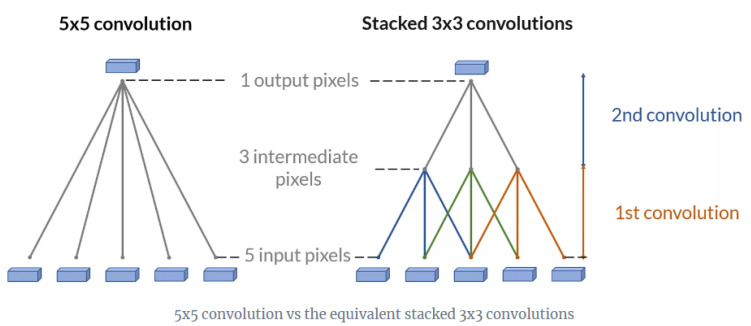
If an input has 5 pixels, a stacked 3 × 3 convolutions reaches the same number of output pixels as a single 5 × 5 convolution. However, it requires only 18 variables to be trained (3 × 3 × 2) instead of 25 (5 × 5) [60].

In 2014, two important CNNs were presented, VGGNet and GoogLeNet. VGGNet was implemented by Simonyan and Zisserman [59]. They investigated how the accuracy of CNN model is related to their depth. The authors presented several CNN models that would vary their layers from 11 to 19. Their models would have similar architecture to AlexNet and LeNet, however as depicted in Figure 5, they used three stacks of 3 × 3 convolution layers instead of one 5 × 5 or 7 × 7 convolution layers, their convolution stride is 1 and the images were padded with zeros to retain its original dimensions. These changes had many advantages, they made the model simpler to implement, they enabled the model to acquire more complex and discriminative features by using smaller receptive fields and more rectification, and made the computation load more efficient since the number of weights (parameters) were decreased. Conversely, the model required a large memory size to store the layer’s parameters for the back-propagation process and the model took 2 to 3 weeks to train one single network. The authors concluded that image classification accuracy was improved when using a deeper CNN network, for example, their VGG 19 had the best results compared to VGG 11 and 16 and it was ranked the second in the ILSVRC14 contest.

GoogLeNet was implemented by Szegedy et al. [61] and their goal was to make a deeper CNN but keeping the computation efficient. This was achieved by constructing an efficient inception module block that was repeatedly used in the CNN. The inception module uses 1 × 1, 3 × 3 and 5 × 5 convolution kernel and a 3 × 3 max pooling. Note that before applying the 3 × 3 and 5 × 5 convolutions a 1 × 1 convolution is applied to reduce the previous layer dimension. This decreases the computation cost and increases the use of the rectified linear activation (addition of non-linearity model). GoogLeNet has 22 layers, it does not have a fully connected (FC) layer and it only has 5 million parameters, which is a huge reduction compared to AlexNet that has 60 million parameters. The CNN architecture was the winner in the ILSVRC 2014 contest with a test error rate of 6.7%. Although GoogLeNet was the winner, it deviated from the classic CNN architecture making the model more complex. In addition, the use of 7 × 7 convolution in the first layer may reduce the feature maps which can affect the performance in the later layers.

GoogLeNet was an important work since the model had a considerable deep CNN of 22 layers and inspired other authors to implement deeper models, however, a problem called degradation surged when the number of layers were increased. For example, He et al. [62] experimented to compare the performance of an 18 and a 34 layers model. The experiment showed that the training and validation error for the 34 layers model was higher than the 18 layers model. To solve the degradation problem, the authors presented a residual learning block. The idea is to directly copy the input X to the forward layers in the neural network, other terms used are skipped connections or shortcuts. By using several residual blocks, the authors were able to create a 152 layers CNN called Residual Network (ResNet) without compromising the training and validation error performance. Resnet achieved a test error rate of 3.57% and it was the winner in the ILSVRC 2015 contest.

**Figure 6:**
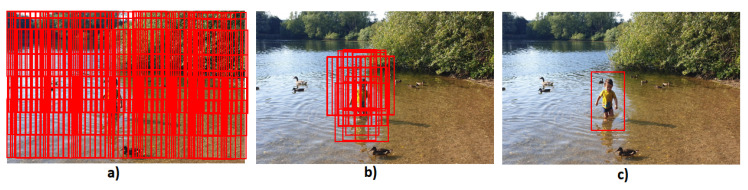
(**a**) Sliding window approach densely applied in all location of the image. (**b**) Only the bbox that have 50% of overlap with the object (child) are used by the regression algorithm to predict the final bbox. (**c**) Predicted bbox are drawn around the object.

Apart from object classification, there is research on object localisation and detection. For instance, the AlexNet model was the winner in ImageNet 2012 contest, both for classification and localisation, however, the authors did not publish the methods used for the localisation procedure. The first work published explaining localisation was done by [63]. The main concept of object localisation is to use a CNN that has two heads, one usually called classifier head and the other regression head. The regression head can be located after the convolution layers or after the fully connected layers. Sermanet et al. [63] placed the regression head in their Overfeat model after the 5th layer and they used a classifier similar to AlexNet architecture. The regression head was used to predict the coordinates used to draw a bounding box around the detected object. As depicted in Figure 6, to get the final predicted bounding box the OverFeat network would be densely applied in all locations and scale of the input image using the sliding window approach. Each sliding window would give a confidence score of the objects category and a bounding box. The bounding boxes that have 50% of overlap with the object would be merged, accumulated, and used by the regression algorithm to predict a final bounding box. The detection task is almost the same as the localisation, the only difference is that the model would need some negative samples to distinguish between background and objects, in addition, the image will have more than one object to be recognised. Overfeat was the winner in the ILSVRC 2013 contest for localisation and detection, achieving top 5 error rate of 29.9% and mean average precision (mAP) of 24.3%, respectively.

**Figure 7:**
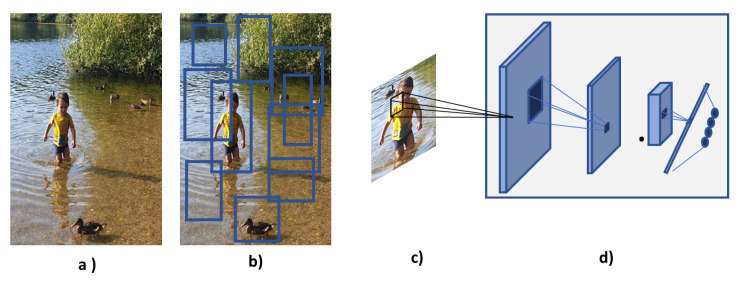
R-CNN architecture: (**a**) Input Image. (**b**) Region proposals are acquired using selective searching technique. (**c**) The regions of interest are warped or cropped and used as the input to the CNN. (**d**) CNN extract features and classify the proposed regions of interest [64].

The sliding window approach used in the Overfeat CNN is very computationally expensive and, it is not efficient since too many windows are used and in most of them there will not be an object. Girshick et al. [64] proposed a system to overcome these issues where they combined region proposal algorithm with CNN. The system generates regions that are likely to have an object using the selective search technique, these proposed regions are then passed in a pre-trained CNN to extract feature vector. Finally, the extract feature vector is fed into an SVM classifier, and a localisation regression approach is used to classify the objects and draw a bounding box around them. The system is called Region Convolutional Neural Network (R-CNN) and it was able to achieve an mAP of 53.7% in the PASCAL VOC 2010 dataset and it outperformed the Overfeat algorithm in the 200-class ILSVRC2013 detection dataset. Although, R-CNN reduces the number of regions per image to be classified, to approximately 2000, this number still requires a large storage disk to cache the extracted features and affects the time taken to train and test the network. The time taken to train 5000 images is approximately 2.5 GPU-days and the time taken to test one image is approximately 47 s which does not meet a real-time system requirement [65]. Other disadvantages are that R-CNN requires that input images have fixed size (224 × 224) because of the fully connected layers; it does not enable-shared computation because it requires one feature map for each sub-image; it is a multi-stage pipeline as it must train three different models, one for feature extraction, one for object classification and one for bounding box regression. Figure 7 depicts the overall architecture of the R-CNN framework.

**Figure 8:**
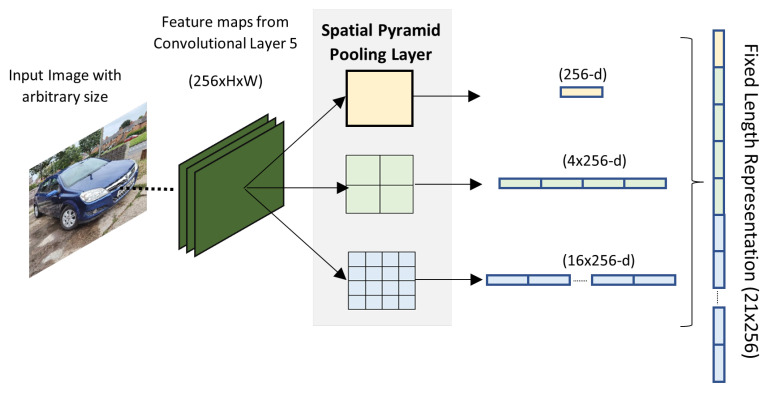
Spatial Pyramid Layers and the output vectors [66].

Because R-CNN algorithm only accepts fixed input images, it needs to perform cropping or warping in the sub-images to acquire a fixed-length descriptor for training. This causes distortion and deletion of the objects parts which yields less detection accuracy. In order to enable networks to accept arbitrary image size/scale He et al. [66] proposed the Spatial Pyramid Pooling Network (SPP-Net). As depicted in Figure 8, the SPP-Net applies a 3 level spatial pyramid pooling on the 5th convolution layer which has 256 feature maps. At level zero a 1 × 1 max pooling is applied in each feature map and outputs a 1 × 256 vector, at level one a 4 × 1 max pooling is applied outputting a 4 × 256 vector, and at level 2 a 16 × 1 max pooling is applied outputting a 16 × 256 vector. The output vectors of each pyramid level are concatenated yielding a final output vector of 21 × 256 which is fed to the fully connected layers. The SPP-Net technique makes output vector dimension independent of the input image size/scale, it is only dependent on the number of feature maps. The SPP-net also has the benefit of the feature maps of the entire image being computed only once. Overall SPP-Net improves both detection accuracy and efficiency. On the other hand, it is still a multi-stage pipeline and since the 5th layers are reused, they cannot be fine-tuned, hence there is a drop in detection accuracy.

**Figure 9:**
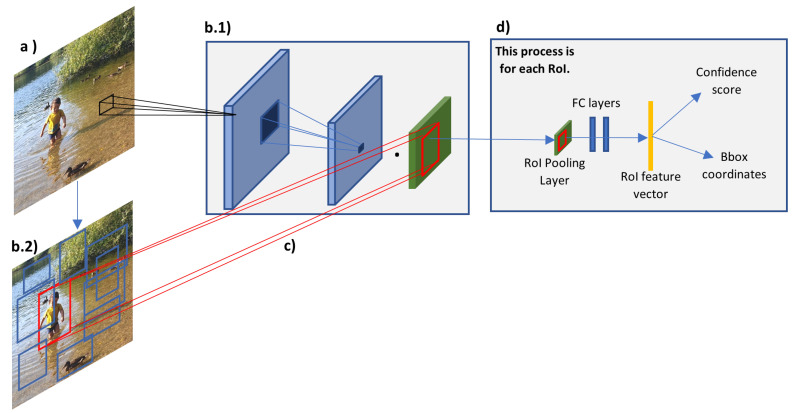
Fast R-CNN architecture: (**a**) Input image. (**b.1**) CNN is used to generate feature maps of the entire input image only once. (**b.2**) RoIs are acquired using selective searching technique. (**c**) Each RoI is projected onto the generated feature maps. (**d**) Pooling and fully connected layers are applied onto the feature maps with the projected RoI to generate a feature vector. The feature vector is used by softmax, and bbox regressor to output confidence score and bbox for each RoI [65].

Girshick [65] presented a second version of the R-CNN, called Fast R-CNN where they were able to increase the mAP, reduce the train and test time, convert the system from a multistage pipeline to a single stage, and eliminate the need for disk storage. As depicted in Figure 9, the main difference between the R-CNN and Fast R-CNN architecture are that the latter one processes the entire image, instead of each region proposal, to create a high-resolution convolution feature map. This enables shared computation (share convolution features), since all the region of interest (RoI) in the image proposed by the selective searching algorithm, can be projected in this high-resolution convolution feature map. Once the RoI is projected into the feature map, the network adopts the SPP technique with only one pooling level to generate a fixed-length feature vector. The feature vectors undergo by few fully connected layers and at some point, the fully connected layers are divided into two parallel processes, one is the SoftMax, to classify the objects categories and the image background, and the second is the bounding box regressor that calculates the numbers required to draw the bounding box around each detected object. The authors achieved a single-stage pipeline by using multi-task learning. The Fast R-CNN takes around 9.5 h to train and 2.3 s to test an image, this is a noticeable improvement compared to the R-CNN system which takes days to train and 47 s to test an image. However, 2.3 s still does not meet real-time system requirement. The bottleneck of the fast R-CNN test time is the time taken by the selective search algorithm to find the region proposals, which takes 2 s. Another disadvantage of the system is that, it is not learning what is the best region proposal, instead, it uses a fixed output generated by the selective searching algorithm.

**Figure 10:**
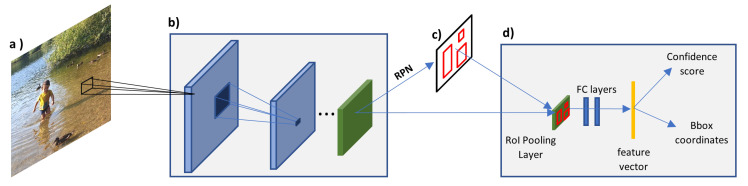
Faster R-CNN architecture: (**a**) Input image. (**b**) CNN is used to generate feature maps of the entire input image only once. (**c**) RoIs are acquired using Region Proposal Network (RPN). (**d**) The RoI is projected onto the generated feature map of the whole image. Then, pooling and fully connected layers are applied onto the feature map with the projected RoI to generate a feature vector. The feature vector is used by softmax, and bbox regressor to output confidence score and bbox coordinates [67].

A system called Faster R-CNN was implemented by Ren et al. [67] and its main contribution was the introduction of Region Proposal Network (RPN) in the Fast R-CNN system. This almost eliminates the time taken for the region proposals stage and enables the system to learn what is the best region proposals. As depicted in Figure 10, the system is almost the same as the Fast R-CNN, but instead of using the region proposals determined by the selective search algorithm, the system uses the high-resolution feature maps as the input to the RPN to determine the regions of interest of the image. The Faster R-CNN system can test an image in 0.2 s (5 fps) and have improved detection accuracy, however, it still requires intensive computation and does not meet the criteria of a real-time system. The system is also more complex because two NNs must be trained, and, somehow, they are dependent on each other, this makes the system complicated to optimise [68].

**Figure 11:**
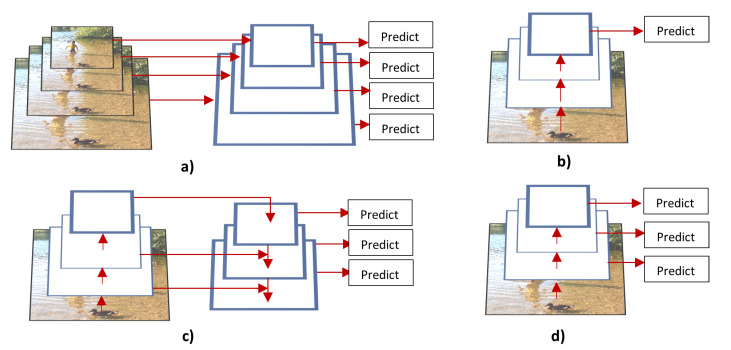
Different types of strategies to deal with scale variance. (**a**) Featurized image pyramid. (**b**) Single feature map. (**c**) Feature Pyramid Network. (**d**) Pyramidal feature hierarchy [69].

Although the mentioned algorithms give acceptable accuracy in the COCO and ImageNet datasets, they struggle to detect objects that are small and in different scales. Figure 11 depicts some of the proposed techniques to tackle these issues. Figure 11a depicts the image pyramids approach, it provides rich semantic information on all scale levels, however, it is not efficient because it requires more memory and time. Figure 11b depicts the single feature map approach which uses only one input scale to generate high semantic information during the training, but during test time, it generates image pyramids. This approach uses less memory and it is quicker, however, the train and test time inference is not consistent. Figure 11d depicts pyramidal feature hierarchy, it treats the different pyramidal feature hierarchies generated by the CNN as a feature image pyramid. This presents a gap in the overall semantic information because high-resolution maps have low-level features. To overcome these limitations Lin et al. [69] further improved the Faster R-CNN network by adding to it a Feature Pyramid Network (FPN). As depicted in Figure 11c, the FPN uses bottom-up, top-down, and lateral connection approaches to deal with the scale invariance problem. These approaches enable the system to merge low-resolution, semantically strong features with high resolution and semantically weak features. In addition, it does not require more memory and time than the latter approaches.

The previous mentioned object detection algorithms, for instance, R-CNN, Fast R-CNN, Faster R-CNN and SPP, are considered two-stage systems. Because first, they calculate the bounding boxes using region proposal and then perform object classification. One-stage algorithms also have been proposed where it combines the detection and localisation into a regression problem. For instance, the You Look Only Once (YOLOv1) algorithm performed by Redmon et al. [70] has unified the object classification and the bounding box as a regression problem. Instead of using region proposal or sliding window, YOLO divides the input image into *SxS* grid cells, then each grid cell predicts B bounding boxes and the confidence score that an object is present in them. If the confidence score is greater than the set threshold, the algorithm will predict the confidence score for each C object-specific class. There are many advantages in this approach, for example, the algorithm is much faster than the Faster R-CNN (state of art algorithm at that time), the algorithm commits fewer background errors since it sees the large context, it learns more general representations of the object which makes the system more robust when tested with new inputs. However, the algorithm has a lower mAP and recall, compared to Faster R-CNN, it has a considerable localisation error number, and difficulties in detecting small objects. As the algorithm divides the input image into SxS grids and each grid can only classify and localise one object, it cannot detect more than 49 objects.

Single Shot Multi-box detection (SSD) is another single-stage system, it was presented by Liu et al. [68]. Their main goal was to perform object detection using a single deep neural network (DNN), that removes the need to re-sample pixels or features to hypothesise bounding boxes but still yields good object detection accuracy. Their network has two parts, one called “base network” and the other called “auxiliary structure”. The base network has similar architecture to VGG-16 network, and it is used for image classification. The main difference between SSD and YOLOv1, is the auxiliary structure in the SSD algorithm. It enables multi-scale features (MSF) maps, pre-computation of the object category score and the bounding boxes offsets by using small receptive kernels (3 × 3 × p, where p is the number of channels). Additionally, several default bounding boxes (anchor boxes) with different dimensions, aspect ratios and scales were used in the MSF maps to predict the ground truth box. SSD system became state-of-art in 2016 both in accuracy and speed. It outperformed Faster R-CNN and YOLOv1 by achieving 74.3% mAP at 59 Frame Per Second (FPS) using the PASCAL VOC2007 dataset.

After SSD, Redmon and Farhadi [71] proposed the second version of the YOLO algorithm. They aimed to make it better, faster and stronger to overcome the limitations of the previous model. They made the algorithm better by using batch normalisation, high-resolution input images, adopting the anchor box method, k-mean clustering, direct location prediction and multi-scale training. The algorithm is faster because instead of using the GoogLenet architecture, they proposed their custom network called Darknet which decreases the number of operations from 8.52 billion to 5.58 billion per image. Finally, the algorithm is stronger because they used WordTree method to combine both ImageNet and COCO dataset to train the network. The final YOLOv2 algorithm achieved an mAP of 76.8% at 67 fps using the VOC 2007 dataset. This achievement outperforms previous state-of-art detection algorithms such as Faster R-CNN and SSD algorithms. However, the algorithm still faces the problem of detecting small objects.

**Figure 12:**
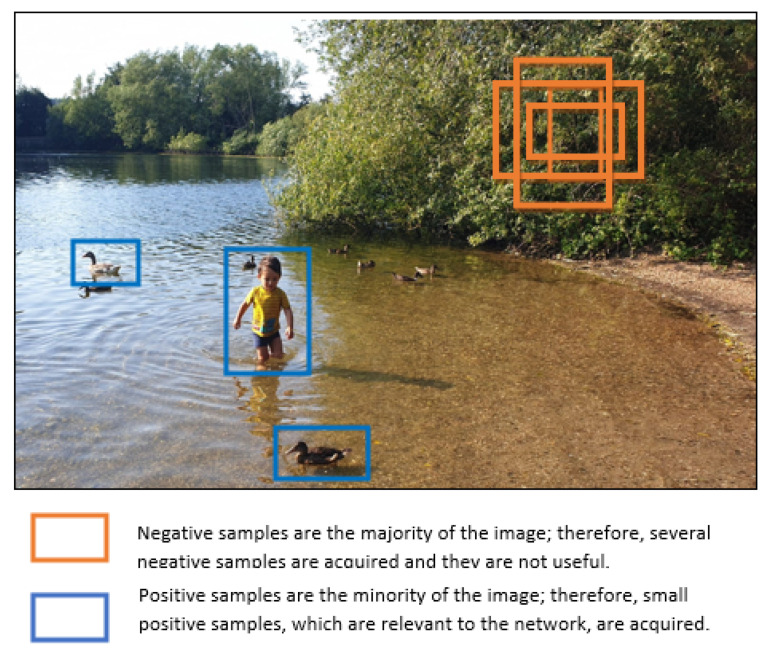
Foreground–background imbalance example, there are more negative (orange boxes) than positive (blue boxes) examples.

By comparing two stage with one stage algorithms, two stage algorithms have better accuracy results, while the one stage algorithms are faster. There was no clear explanation why one stage algorithm has lower accuracy, hence Lin et al. [72] investigated and found that the issue with the one-stage algorithm, which performs dense sampling, is the foreground–background class imbalance. In other words, as depicted in Figure 12, there is more background information meaning more negative samples, than objects, which are positive examples. To overcome this problem, the authors modified the standard cross-entropy loss function, in which the negative samples contribute more to the final total loss results, and presented the Focal Loss. The focal loss function, down-weights the contributions of easy samples and focuses more on hard examples, hence the positive samples contributes more to the total loss result. The authors used this modified loss function and created the RetinaNet network that was able to achieve higher accuracy and faster detection speed than the previous two and one stage algorithms. The disadvantage of the algorithm is that it requires to fine-tune one more hyper-parameter which is the focusing parameter.

Other versions of YOLO are YOLOv3/v4/PP/v5. In YOLOv3 Redmon and Farhadi [73] focused on making the algorithm more accurate by extending their classification Darkenet-19 network to 53 layers, and adopting the concept of residual learning block in their network architecture. To overcome the problem of detecting small objects, the authors predict the bounding boxes at three different feature map scales, 13 × 13 to detect large objects, 26 × 26 to detect medium objects, and 52 × 52 to detect small objects [74]. Zhao and Li [75] and Yang and Deng [76] presented a modified version of the YOLOv3. Zhao and Li [75] instead of using the k-means cluster to determine the height and width of the bounding box priors, which is time-consuming when dealing with high scale variance image datasets, they used Markov Chain method. This change enabled the YOLOv3 to achieve better Average IoU and faster run-time. In addition, the algorithm was able to slightly improve the recall, mAP and F1-Score. Yang and Deng [76] did not modify the convolutional architecture but they combined both of the features extracted by the convolutional layers and features extracted by the FPN to acquire more semantic information. They also proposed global context blocks, which is based on self-attention mechanism, in order to pay more attention to the relevant information of the feature maps. These techniques increased the YOLOv3 algorithm accuracy with a slight addition of computation cost.

In YOLOv4 Bochkovskiy et al. [77] focused on optimising the speed and accuracy of the system, in such a manner that only one conventional GPU is required (e.g., 1080Ti or 2080Ti GPU). In their paper, they described that one stage object detector is made of several elements, input, backbone, neck, and head. Where the input is the image, the backbone is the classification algorithm that is usually pre-trained using ImageNet dataset, the neck is the element where features map of different stages are collected and combined to yield a higher receptive field, which allows the network to learn variance in different images scale and size, and the head is the part where the object classification and bounding boxes are predicted. The authors did a thorough study to identify which algorithm for each element would yield an object detector algorithm with high speed and good accuracy. The final architecture of the YOLOv4 is composed of CSPDarknet53 as the backbone, SPP and PAN as the neck, and YOLOv3 as the head. The algorithm became state-of-art where it achieved an accuracy of 43.5% AP using the MS COCO dataset, and it can process approximately 65 fps, which meets the real-time requirement. The PP-YOLO system was presented by Long et al. [78], their goal was not to introduce a new detector algorithm but use existing tools to create an effective and efficient version of the YOLOv3 algorithm. The main differences between the previous YOLO versions were that they used the ResNet network as the image classifier; they used a larger batch size and Exponential Moving Average (EMA) to make the system more stable; DropBlock to prevent overfitting; and Intersection Over Union (IoU) Loss, IoU Aware, Grid Sensitive, Matrix NMS, CoordConv and SPP to increase the model accuracy. They outperformed YOLOv4 both in accuracy and speed, they achieved 43.5% mAP at speed of 72.9 fps, whereas, YOLOv4 achieved 43.5%mAP at 62 fps. YOLOv5 has been implemented by Jocher [79] but there is still no paper reporting their work. Quang et al. [80] proposed a new single-stage algorithm which has two main modules, the local information extraction model (LIEM) and the global information extraction model (GIEM). The former model uses a bidirectional FPN to extract multi-scale features from different convolutional layers, and the latter is responsible to extract global features from downsized images. Once local and global information is extracted, they are combined using an aggregation network. The LIEM and the GIEM modules were introduced to acquire richer features, since the authors claimed that important information is lost when only the low-level and high-level feature maps are combined. The algorithm was able to improve AP by 1.6% and achieved a detection speed of 45.43 fps when tested with the MS COCO dataset.

## 4. Vehicle Detection

**Figure 13:**
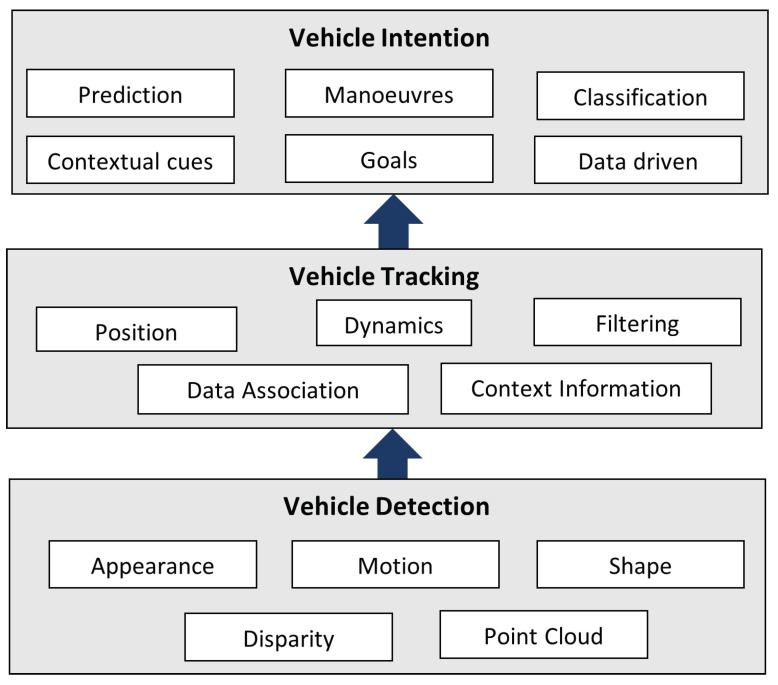
Block diagram to present the stages of vehicle detection, vehicle tracking, and vehicle behaviour. The vehicle detection is performed using appearance, motion, shape, point cloud, and disparity techniques. Vehicle tracking is acquired using position, dynamics, filtering, context information, or data association. The vehicle behaviour is determined using manoeuvres, contextual cues, data driven, or goal-orientated approaches [11].

Vehicle detection is a key stage for many of the Intelligent Transportation Systems (ITS) such as AV, ADAS, Traffic Surveillance and Traffic Statistics. As depicted in Figure 13, vehicles must be first detected to then perform vehicle tracking and vehicle behaviour prediction. A vehicle detection system should be robust, fast, accurate, and at low cost. Different sources of images are used for vehicle detection, for instance, images from traffic surveillance cameras, cameras mounted on vehicles, UAV cameras and satellites images.

This research will only focus on on-road vehicle detection, where the camera is mounted in the vehicle. The main challenges for a vehicle detection system are:

- Vehicle can belong to many classes (e.g., car, bus, truck, etc.).
- Vehicles belonging to the same class have varieties of shape, structure, colour, and size.
- Vehicles are viewed in different orientations (e.g., side-front view, side-back view, left-side, right side, etc.).
- One image might have several vehicles in different scales. Large size and small size vehicles have scale-variance, for instance, different visual characteristics and feature maps.
- Vehicles are more prone to be cluttered in complex traffic scenes.
- Vehicles are in environments that are dynamic due to different weather conditions (e.g., sunny, rainy, foggy and snow.), different times of the day (e.g., day, dusk, and night) and passing through tunnels. These factors affect the image background and illumination.
- For on-board vehicle detection, it is necessary to take into consideration the ego and target vehicle motion [21].

Currently, in order to overcome these vehicle detection challenges, traditional and DL techniques are used. These techniques generally are a two-stage processes, the first stage is to extract RoI candidates, and the second stage verifies if the generated candidates are the objects of interest.

### 4.1. Traditional Techniques

For detailed literature reviews on traditional vehicle detection, tracking and behaviour prediction algorithms please refer to [11,12,13,14,15,21,81]. Traditional vehicle detection is mainly performed by motion-based and appearance-based approaches. Motion-based approaches have been implemented using optical flow techniques, such as dense optical flow, sparse optical flow or Scene Segmented Establishing Tracking (ASSET-2). However, the performance of these techniques is affected by camera movement/vibration, they are not able to classify the different objects in motions, they are limited to detect slow motion vehicles, several images are required to detect object motion, and many post-processes are required to refine the results, which makes the algorithm computationally expensive and complex [82]. The appearance-based approach uses basic or advanced features extracted directly from the pixel image. Basic features used in the literature are corners, colour, symmetry, texture, vehicles shadow, vehicle’s headlights and taillights, edges, and so forth. However, these basic features are easily affected by reflection, low light intensity, different weather conditions and external objects. In addition, only one of these basic feature is not enough to describe all the rich information given by an image, hence it was proposed to use multiple of these features, but this makes the system more computationally expensive and complex. Advanced features are extracted using algorithms like HOG [83,84], Haar-Like (wavelets) [85], combination of HOG, Local Binary Patterns (LBP), and Haar-like [86], SIFT [87], PCA [88], SURF [89], And-Or model [90], and Gabor filter [91]. Once features are extracted, discriminative classifier algorithms such as SVM, ANN, Mahalanobis Distance, or AdaBoost are used to classify the objects as vehicle or non-vehicle. Generative classifiers like probabilistically weighted vote, hidden Markov models and Gaussian mixture models are also used in the literature; however, the discriminative approach is preferable, since it gives a distinct classification of vehicle or non-vehicle, instead of a distribution probability for each object class. Although the above extraction and classification algorithms have shown acceptable results for vehicle detection in simple traffic scenes, their performance is limited to complex traffic scenes, as they produce too many false-positive results and they are still highly affected by occlusion, illumination, scale sensitivity, and background environment [82].

### 4.2. DL Techniques

Recently, to overcome these challenges, researchers have been investigating the use of DL techniques, since they can learn what are the best features to extract in order to detect vehicles. This section will present some of the most relevant on-road vehicle detection works between 2016 to 2020, that adopted DL technique. Based on the reviewed works from Table 4 the research community have tried, either to improve vehicle detection robustness and accuracy or efficiency. The following approaches were used to improve detection **robustness**: extract and retain more discriminative information from the input image, handles scale sensitivity, fine-tune existing generic object detection algorithm, and handles occlusion and truncation problems.

The following works aimed to extract or retain more discriminative information from the input image. To the best of the author knowledge, one of the first DL works for vehicle detection, was performed by Wang et al. [92], where they used 2D deep belief network (2D-DBN) to extract more discriminative information, which made the system more robust to complex scenes and achieve better results than the traditional techniques. However, the datasets used are very basic and do not take into consideration occluded and multi-scale vehicles.

**Figure 14:**
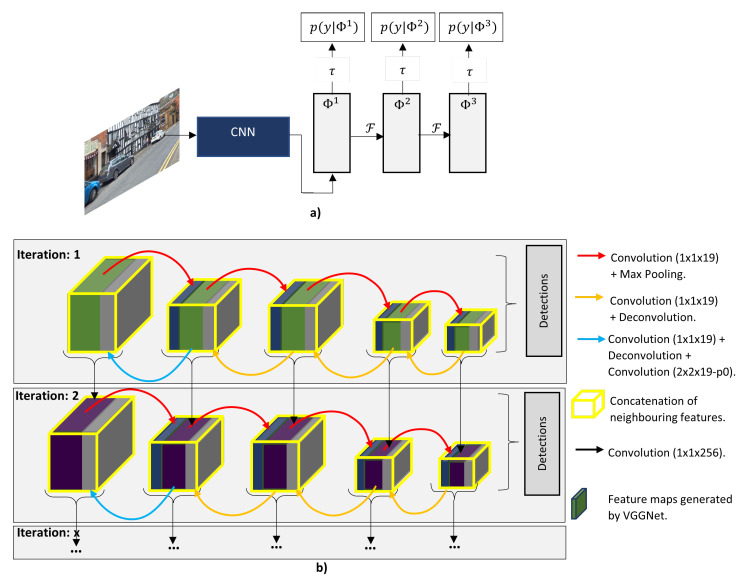
Recurrent Rolling Convolution (RRC): (**a**) The recurrent component of the RRC, where I is the input image, Φ is the feature map and F is feature aggregation function. This block is considered recurrent because the weights for F and τ are shared in each step, for instance from Φ1 to Φ2 and to Φ3. (**b**) It is considered the rolling component because it performs downwards and upwards feature aggregation [101].

Liu et al. [102] argues that algorithms such as R-CNN have difficulty to detect small objects, as the feature maps used for region proposal have limited discriminative information, therefore, they proposed the Backward Feature Enhancement Network (BFEN), which adds discriminative information from high levels layers to low-level layers in order to acquire high-quality region proposals. The generated region proposals are then refined using the Spatial Layout Preserving Network (SLPN) which replaces the conventional FC layers with the split-transform-merge (STM) blocks. The BFEN have the advantage to increase the recall values for small objects.. Ren et al. [101] presented a novel DL network called Recurrent Rolling Convolution (RRC) to improve the mAP of a single-stage detector. As illustrated in Figure 14 the RRC have the recurrent and the rolling components, these components enable the system to perform feature gathering and aggregation to extract more contextual information. RRC has the best result in the KITTI moderate and hard categories for the 2D monocular vision system.

Hu et al. [113] proposed a cascade vehicle detection algorithm which combines a traditional detector and DL classifier. The traditional detector uses HOG, LBP, and Haar Like to extract features and SVM to classify vehicle/no-vehicle. Since traditional detectors in complex scenarios have high false positives, the DL classifier uses the output of the traditional detector to further enhance the classification performance.

The following works aimed to handle scale sensitivity problem [82,94,96,112]. Cai et al. [94] implemented a network called multi-scale CNN (MS-CNN) where they were able to detect vehicles at different scales by using information from different features maps resolution. The MS-CNN network is made of a proposal and a detection sub-network. At the proposal sub-network the main convolutions layers streams are divided into three other branches in order to create three detectors. These three detectors are then combined and used by the detection sub-networks to yield a final multi-scale detector. The algorithm performed well on the KITTI easy and moderate categories, but it falls behind in the hard category.

The SDP+CRC(ft) implemented by Yang et al. [96] uses Cascade Rejection Classifiers (CRC) to reject easy negative samples (background). The regions that are not rejected are evaluated by the Scale Dependent Pooling (SDP) model. If the region is small then the SPD module pools the low-level convolutional features. On the other hand if the region is large then the SDP pools the high level convolution. The algorithm was able to improve AP and make the detection more efficient.

**Figure 15:**
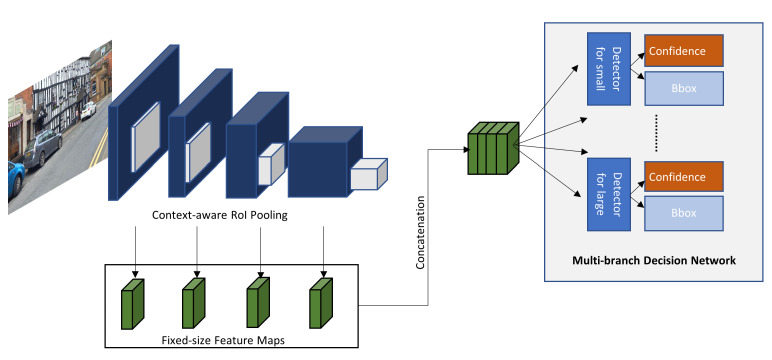
SInet: The blue and grey blocks are the feature maps and the proposed RoI, respectively, at different scales. The separated green blocks are the fixed size feature map, pooled by the context-aware RoI pooling for each RoI scale. The fixed size feature maps are then concatenated and used by the multi-branch decision network to classify the objects [82].

Hu et al. [82] identified that the scale sensitivity problem in DCNN has to do with the RoI pooling method that does not conserve the features of small objects. They also identified the large intra-class distance between small and large scale images of an object that belongs to the same class. To overcome these issues, they presented the DCNN, depicted in Figure 15, called Scale-insensitive Convolutional Neural Network (SINet) that uses context-aware RoI pooling to conserve features of small objects and a multi-branch decision network to deal with the intra-class distance. Even though, SINet has a slightly lower performance than the other cited methods from Table 4, they have the best detection speed of 0.11 s and 0.2 s.

Wang et al. [103] presented an Adaptive Perceive SSD (AP-SSD) network. The network replaces the low-level convolutional kernel with multi-shape and colour Gabor filters to improve detection accuracy. It also has a dynamic region enlargement module, that uses the Accuracy Gain (AG) map generated by the Amplified Precision Gain regression Network, to zoom in at specific regions of the input image where small objects are located. These region candidates are then fed into the SDD detector. The authors implemented the detector with an LSTM network to further improve accuracy and to enable tracking. The LSTM enables the information of feature maps to be shared at different frames.

**Figure 16:**
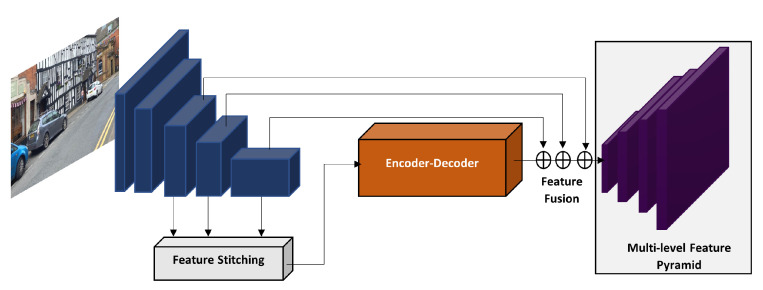
Multi-level FPN YOLO: three convolutions layers at different levels are stitched, then this stitched features are encoded and decoded. Finally, the original convolutional layers that were used by the stitching component and the decoded stitched features are fused to yield a multilevel feature pyramid [112].

Hong et al. [112] modified the YOLOv3 algorithm by presenting a new multi-level feature pyramid (FPN). As illustrated in Figure 16, the multi level feature pyramid has three modules, the feature stitching, encoder–decoder and the feature fusion. The network was able to achieve impressive results on the KITTI dataset, beating all the mono camera vehicle detection works. It only stays behind the works that use 3D cloud points, which are out of the scope of this paper. However, its detection speed of 2.1 s is too high and their algorithm has not been submitted at the KITTI benchmark.

Fan et al. [93], Gao et al. [98], Wang et al. [105], Fan et al. [111] noticed that Faster R-CNN [67] has a good vehicle detection performance on dataset such as PASCAL 2007, but not so well on the KITTI dataset which has more occluded, different angle view, and multi-scale vehicles. Therefore, by fine-tuning the Faster R-CNN algorithm they were able to achieve good results on the KITTI easy and moderate categories but not so well on the hard. Chu et al. [99] improved vehicle detection robustness by presenting a novel multi-task learning and ensemble DCNN called Region of Interest Voting CNN (RV-CNN) and a novel RoI voting method. The algorithm was able to beat all the other mono camera works in the KITTI hard category at that time.

**Figure 17:**
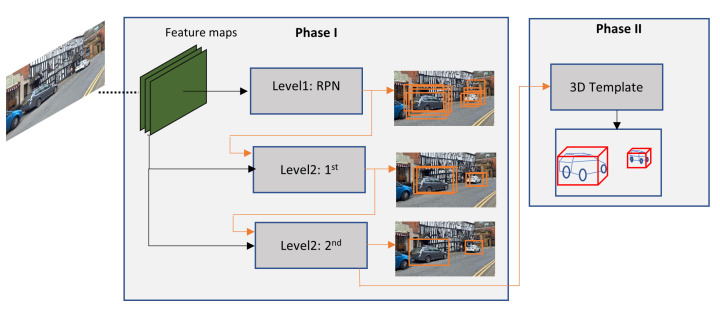
DeepMANTA: at phase I three level of refinements is applied; and at phase II the predicted bboxes from Phase I are used to infer 3D coordinates [100].

3D information from the objects is valuable because it can help to deal with occlusion and truncation problem. Therefore, some authors presented some techniques to enhance vehicle detection by extracting 3D information from monocular camera, for instance, Chabot et al. [100], Bao et al. [107], Jörgensen et al. [108]. Figure 17 depicts a DCNN presented by Chabot et al. [100] called Deep Many-Tasks (DeepMANTA). DeepMANTA have three-levels of refinements, the first level is the generation of RoI, then the second level uses these generated RoI to predict a set of bounding boxes, and finally, the predicted bounding boxes are refined again to yield a final set of bounding boxes. This technique, helps to deal with large intra-class distance problems. The last stage of the DeepMANTA is to use the final predicted bounding boxes to infer 3D information of the vehicle. At the time DeepMANTA became the state-of-art mono camera vehicle detection system, achieving results of 96.40%, 91.10% and 80.79% AP in the KITTI easy, moderate, and hard categories, respectively. The main drawback of the algorithm is its detection speed that ranges between 0.7 s and 2.0 s.

The monocular Feature Enhancement Networks (MonoFENet) algorithm was introduced by Bao et al. [107], it has two main parts. The first part uses VGG-16 or ResNet-101 to extract features, it adopted the RPN from the faster R-CNN and RoI max pooling to proposal object categories and 2D bounding boxes. The second part uses the Deep Ordinal Regression Network (DORN) to estimate disparity from the input image, then RoI mean pooling is used to create RoI point clouds, then PointFE Network is used to extract features from the point clouds. Finally, the features acquired from the image and the point cloud are combined to estimate 3D localisation. Jörgensen et al. [108] presented the Single Stage Monocular 3D (SS3D) which uses ResNet-34 or Dilated Residual Network (drn_c_26) to encoder features and non-linear least squares optimiser to estimate 3D bounding boxes. To overcome occlusion, Zhang et al. [106] presented a Faster R-CNN detection system that uses part-aware RPN to capture both global and local information from the vehicles. The algorithm outperforms DeepMANTA in the KITTI hard category; however, its detection speed is still high 2.1 s.

The following works aimed to improve vehicle detection **efficiency**. Yuan et al. [97] present, a graph-based algorithm that decreases the number of RoI candidates compared to the traditional sliding-widow approach, it achieved a detection speed of 1.57 s (2.0 GHz Xeon CPU), however, it does not meet the real-time requirements. The MS-CNN, SDP+CRC(ft), and SS3D works not only aimed to improve robustness but detection speed too. The MS-CNN applied feature up-sampling instead of input image up-sampling using deconvolution and managed to keep the detection speed to 0.4 s (Intel Xeon CPU 2.4 Hz and NVIDIA Titan GPU). The SDP+CRC(ft) algorithm improved detection speed using the CRC to reduce the total number of region proposals that will go under feature extraction and achieved a detection speed of 0.6 s (NVIDIA K40 GPU). The SS3D achieved a detection speed of 0.048 s (Tesla V100) by building a lightweight CNN, estimating the bounding boxes for each proposed object independently, and in parallel, and using the input image only once during the feature extraction stage. Chen et al. [110] presented a DCNN based on the DenseLightNet aiming to decrease the resources requirements and increase detection speed. The algorithm was evaluated using the PASCAL 2007 dataset and it only uses 10.1 MB of memory and achieved a detection speed of 71 fps (GeForce Titan X GPU). However, it only achieved an average precision (AP) of 82.5%.

**Figure 18:**
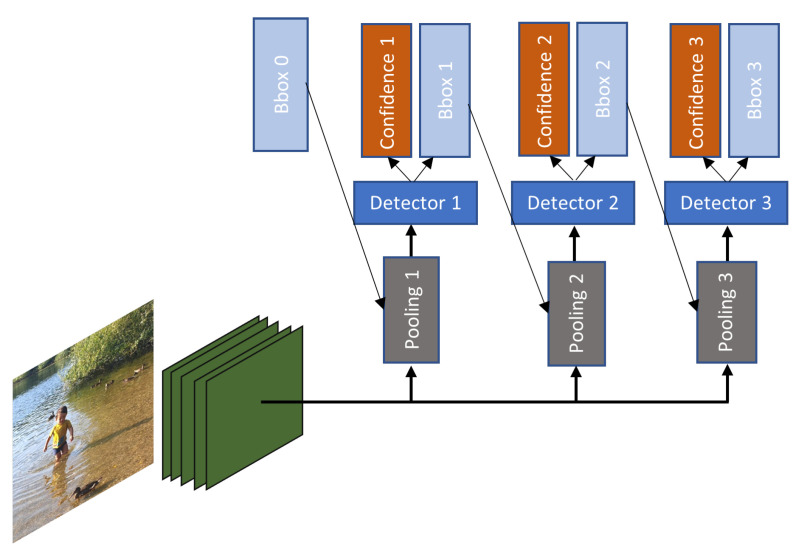
Cascade R-CNN: an input image is convoluted with the backbone convolutions layers (green), then three different pooling (grey) are applied in the convoluted image. Each one of the three pooled feature maps are fed into different detectors(dark-blue). The detectors then predict the class of the objects(orange) and their respective bounding box(light-blue). Notice that the predicted bounding box "bbox X" from the previous detector is fed as input to the next detector. This cascade approach enables the network to train with a higher IoU threshold which increases the detector quality [104].

Generally, the studies are more focused on improving accuracy, robustness, or efficiency. However, Cai and Vasconcelos [104] proposed a network called cascades R-CNN, with the aim to improve detection quality. As depicted in Figure 18 several detectors, in this case Faster R-CNN, are used to perform re-sampling mechanism, where the output of the first stage detector is fed as input to next stage. Cascades R-CNN has increased AP but it takes more time to train and test as the number of stages increases. However, this time overhead it is not high, because only the regression operation is computed which is much less than the feature extraction computation.

## 5. Pedestrian Detection

Pedestrians are considered one of the most vulnerable road users, therefore, AVs must be able to detect them in order to avoid collisions. For this, AV has to detect pedestrians with high accuracy and low inference time. This section is not intended to present a literature review on pedestrian detection, but it will present an overview of the pedestrian detection challenges and techniques used over the years. For literature reviews please refer to the following works, [6,7,114,115,116,117,118,119,120,121]. Pedestrian detection is considered a challenging task for the following reasons:

- Inconsistence of pedestrian appearance, for example, pedestrians wear different types and colours of clothes, have different heights, carry different objects in their hands, and constantly change their pose;
- They are more difficult to detect in environments that are cluttered (busy urban areas), have a high variance of illumination, are very dynamic and have poor weather conditions;
- One image might have several pedestrians in different scales. Pedestrian that are far away in the image does not have distinct boundaries and are obscure;
- Large size and small size pedestrians have scale-variance, for instance, different visual characteristics and features maps [122];
- Pedestrians change directions very quickly.

The main components used to tackle the challenges of pedestrian detection are, feature extraction, part deformation model, occlusion model and classification algorithm [123]. The feature extraction component uses algorithms to extract distinct features (descriptors) from an input image that describes a pedestrian, for example, shape, colour (greyscale or CIE-LUV), motion, edges, texture and gradients [114]. The extractor algorithms can be either holistic, where it will look for features that describe an object as a whole (e.g., full pedestrian); or part-based, where it will look for features that describe different parts of the object (e.g., pedestrian head, trunk, and limbs). The features extractor algorithms, are usually categorised as background subtraction, appearance, or motion-based. Background subtraction involves the subtraction of two given images to isolate moving objects. This technique is more suitable for surveillance systems because the camera usually needs to be static, and for systems that use binocular stereo sensors. In the appearance approach, the features are extracted directly from the image or video pixels. Optical flow detects velocities of movements from moving objects in an image. These velocities movements are then represented as brightness patterns [124]. Part deformation model is the component responsible to handle the pedestrian’s articulations such as head, trunk, and limbs. Part deformable models are suitable to handle occluded objects, but its performance is highly affected when applied to objects that are too small and with low resolution. One state-of-the-art deformation model algorithm is the Deformable Part Model (DPM) [125,126]. It was previously discussed that pedestrians can be easily occluded by other objects, hence an occlusion handling algorithm such as the detection scores of blocks or parts [127] is used. The classification algorithms use the information acquired from the extracted features to classify the objects as pedestrian or non-pedestrian.

In the literature, pedestrian detection has been implemented using either traditional computer vision, DL, or hybrid techniques. The hybrid technique combines traditional and DL techniques.

### 5.1. Traditional Techniques

Dalal and Triggs [47], Viola et al. [128] are considered the pioneers of traditional pedestrian detection, because of their ground breaking work for the pedestrian detection system. Some of the most remarkable traditional pedestrian detection works from 2000 to nowadays are listed in Table 5, for more works between 2000 and 2015 see [6].

Table 5 describes what feature extractors and classifiers the authors have used, as well as their miss rate (MR) percentage in the Caltech dataset [6]. According to the table, most of the works adopted the combination of HOG and linear SVM or HOG and AdaBoost as the baseline detector. Then, from the baseline detector, the authors tried to improve the detection accuracy by increasing and diversifying the features. Another trend from the table is that as the numbers of extracted features increased the better the MR percentage was got, for instance, the Katamari-V1 algorithm used HOG, LUV, DCT and optical flow features and achieved an MR of 22.49%, and the FSSS algorithm used ACF (HOG+LUV+Normalised gradient magnitude) and Feature selected self-similarity and achieved an MR of 13.93%. These trends were also noticed by Benenson et al. [6], where they concluded that: choosing the right training dataset can improve system performance, the classifier does not play an important role in order to improve the detector quality, the multi-scale system only have a small contribution to the system accuracy, DPM might not be necessary for detection in normal conditions, but it may handle occlusion problems, image context features improve accuracy, using additional information such as optical flow and finally, stereo images might improve the system; however, some of the top detection systems only use appearance information.

### 5.2. Hybrid and DL Techniques

As observed in Table 6, from 2015, the number of works submitted at the Caltech benchmark using traditional techniques started to decrease. In contrast, as reported in Table 6, from 2015 to 2017 the hybrid technique was heavily researched. It is observed that hybrid algorithms that had more extracted features were the ones with the best MR percentage, for instance, the MCF and SA Fast-RCNN algorithms achieved MR of 10.40% and 9.32%, respectively. Although hybrid techniques have achieved impressive results from 2017 to nowadays, most works adopted the DL technique. One reason for this preference is that DL techniques achieved similar or better results and does not require handcrafted features. The types of DL networks used for pedestrian detection are CNN, Recurrent Neural Network (RNN)-Long Short Term Memory [144] (LSTM), Ensemble network, Cascade network, and Encoder-decoder networks. Recurrent neural networks are good for a system that uses temporal or sequential data. It has been used to refine feature maps [101], to learn specific features [145], to improve bounding box localisation [146], and to extract semantic information [147]. Ensemble networks fuse multiple learning algorithms to improve the system accuracy. Du et al. [148,149] fused several DNNs to refine the pedestrian candidates generated by the SSD algorithm, Brazil et al. [150] fused RPN and R-CNN to improve their system performance, and Brazil and Liu [145] fused several RPNs to improve the system precision. As mentioned by Brazil and Liu [145], the limitation of ensemble networks is that they require a lot of memory as the networks become bigger. Cascaded networks also involve multiple learning algorithms; however, they are somehow connected in series. The information learned by one network is transferred to the next network. Brazil and Liu [145] used cascaded networks to improve detection precision. Encoder–decoder networks are a kind of RNN that allows the input and the output sequence to have different lengths. Brazil and Liu [145] used encoder-decoder to compact the information sent from one node to another, this reduces the use of memory. The state of art algorithm in the Caltech benchmark is the AR-Ped where the authors combined all the previously discussed DL networks (Ensemble + Encoder-Decoder + Cascade + RNN) and achieved an MR of 6%.

More recently, researchers have attempted to improve detection algorithm generalisation performance, using cross-dataset evaluation and progressive training pipeline techniques. Cross-dataset evaluation is the process of training and testing the detection algorithm with different datasets. Progressive training pipeline, merges two or more datasets to train the algorithm and then uses another dataset to test it. It also involves the procedure of pre-training, training, and testing the algorithm with a different dataset. For instance, Hasan et al. [151] performed an extensive study, and reported that pedestrian detector generalisation performance can be improved when using cross-data evaluation and progressive training pipeline. In their paper, they trained the detection algorithm with the CityPerson dataset and tested it with the Caltech dataset, and they were able to achieve MR of 8.8%. They also pre-train, trained, and tested their algorithm with the Wider Pedestrian, ECP, and Caltech datasets, respectively, and were able to achieve an MR of 2.5%.

## 6. Discussion

This paper surveys how generic, pedestrian, and vehicle detection algorithms have evolved from simple traditional techniques to DL, and investigates the main limitations of DL object detection algorithms and what has been done up to now to overcome these.

Up until 2008, basic feature extractor algorithms were widely used for classification and detection, however, these features are easily affected by reflection, low light intensity, severe weather conditions and other objects. Hence, from 2008 to 2015 traditional ML algorithms became popular; however, these algorithms are highly affect when tested in complex scenarios and the features are manually extracted. From 2015 to 2017, hybrid approaches were used and showed a good detection performance, but they still need features to be manually extracted. From 2017 to nowadays, most works have adopted DL techniques since they have shown to have better performance than traditional algorithms, require less time and expertise to be developed. However, traditional algorithms also have their advantages, for example, they require less resource, they are not considered as black box, and they might be more useful for systems that do not require generalisation. In the past, DL was restricted to limited CPU, GPU, memory, and dataset resource, but nowadays, as these resources became more available, DL learning has significantly evolved, for instance, a conventional CNN would have only five layers [52] and nowadays there are networks with more than hundred layers [62]. The most used DL technique for object detection are CNNs but RNNs (LSTMs) [101,145,146,147] and ensemble networks [148,149,150] are becoming popular.

Detection algorithms, especially for pedestrians and vehicles, must be accurate, robust, fast, and low cost. One point to clarify is the relationship between accuracy and robustness for detection algorithms. Yang et al. [167] reported that there is a trade-off between robustness and accuracy. However, from the authors’ point of view, the reviewed techniques that were used to improve robustness positively reflected on the accuracy. For example, [82,94,96,112] used techniques to make detection algorithms more robust to scale variance and they reported an increase in accuracy. This relationship also applies if robust techniques are used to detect objects that are affected by occlusion, severe illumination and weather conditions. If this relationship is considered, then to make generic objects, pedestrians and vehicles detection systems more accurate and robust, the reviewed works made use of the following approaches:

- Deeper networks to extract more features from different levels of abstraction. The downside is that the deeper network can suffer vanishing gradient issues, and it requires more memory and computational work [62];
- High-resolution input image approach which enables the network to extract more discriminative information, however, it requires more memory and computation power [56];
- Smaller receptive kernels to extract more information from the input image. However, smaller receptive kernels focus more on local information [59];
- Transfer learning technique, enables a network to transfer the parameters learnt from a task to another. This enables the network to learn in less time and to have better performance. However, the transferring of learning is only possible if the new task is closely related to the previously learned one;
- SSP to avoid warping, cropping, and accept images with different sizes and scales. It might affect accuracy performance, since the layers where the SPP is placed cannot be fine-tuned [66];
- Algorithms, such as WordTree, to combine multiple datasets. This enables the algorithm to improve performance and have better generalisation, but it can be complex to implement [71];
- Use different feature maps to predict bounding-box coordinates for multi-scale objects [73];
- Focal Loss Function to pay more attention to the positive sample (actual object) instead of the negative samples (background). This increases the algorithm efficiency and accuracy, however, it is required to fine-tune one more parameter [72];
- Multi-scale detection approaches such as multi-scale images, multi-scale features, anchor boxes, FPN, or using information from different layers of the network. An ideal multi-scale detector would be able to use various input images with different scales; however, this is very computationally expensive. The other cited options use methods to approximate the multi-scale feature map, therefore, the algorithm might be losing important information that could be used to better detect the objects [94];
- Region proposal algorithms that pay more attention to small objects. This increases the recall values of small objects, but it can be complex to devise [102];
- Multi-branch decision networks to deal with the intra-class distance. It increases the accuracy performance, however, it can be slightly more complex to implement because each branch needs to be designed according to the different scales of the object;
- Multitasking learning enables sharing of knowledge and can increase accuracy performance. However, it slightly increases the computation load [99];
- Ensemble network enable diversification but can make the algorithm computational expensive;
- 3D information, which provides more discriminative features to better detect and localise objects [100,107,108];
- Multiple levels of refinement, which increases the accuracy performance, but increases the detection speed as well [100];
- Disparity and point clouds estimation algorithms. They are used to extract 3D information which can increase the accuracy performance, however, they increase the algorithm complexity too [107];
- Part-aware RPN to increase the robustness of the algorithm to detect occluded objects. The drawback is that, it is complicated to combine the different detected parts to form the whole body [106];
- Techniques to transfer information from high-level layers to low-level layers to acquire high-quality region proposal [102];
- RNNs (LSTMs) to refine detection and localisation;
- RRC to improve accuracy detection and bounding box quality. After six iterations of the RRC, there is no further improvement because it needs an efficient memory mechanism [101].
- Feature fusion techniques to acquired richer feature maps as in Zhu and Wu [161], Wu et al. [164], Shao et al. [165].
- Deformable part technique to detect occluded objects. It increases the detection accuracy; however, a method is required to combine the detected parts of the same objects as in Luo et al. [163], Xie et al. [166]

Pedestrian and vehicle detection systems must be efficient in terms of memory and processing speed, since AVs must detect objects in real-time to plan upfront the actions that it must take. In addition, embedded devices are usually used by AV, although they are smaller and cheaper than actual GPU and CPU, they have limited memory and processing speed. In order to make the detection system more efficient, the reviewed works made use of the following approaches:

- Pooling layers or 1 × 1 convolution kernels to down-sample feature maps [56,61];
- Stacks of small kernels instead of bigger ones to compute less variables [59];
- Share computation where feature maps are computed only once [66];
- Region proposal algorithm or RPN instead of densely apply sliding windows in the whole image [64];
- Feature pyramid instead of image pyramid [69];
- Single stage instead of two stage algorithms, since they do not require region proposal [68,71];
- Decrease the number of RoI candidates [99].

The following paragraphs take into consideration the achieved accuracy and efficiency of the current pedestrian and detection algorithms, in order to discuss if they can be deployed in an AV perception system.

AVs are safety-critical systems and it is crucial that they should detect small and occluded pedestrians and vehicles with a high accuracy. For instance, it was previously mentioned that road accidents are one of the main reasons for children deaths, since they are generally smaller and harder to detect. In a situation where vehicles are at a high speed, drivers must detect pedestrians or vehicles further ahead to take appropriate actions. However, objects that are further ahead are smaller and have low resolution. Pedestrians, especially children, can be easily occluded between parked cars. The review shows that, although many approaches were presented to improve the detection performance of pedestrian and vehicle algorithms, they still struggle to detect small and occluded objects. For example, the two works reported in this paper that acquired the best results for a mono-vision pedestrian on the KITTI dataset were Cai et al. [94] and Brazil and Liu [145], where the former work achieved 83.92%, 73.70% and 68.31%; and the latter achieved 83.66%, 73.44%, and 68.18% for the easy, moderate and hard categories. The work that achieved the best result for vehicle detection reported in this paper on the KITTI dataset was performed by Ren et al. [101], where they were able to achieve 90.19% and 86.97% in the KITTI moderate and hard categories. It is noticed that the detection accuracy drops when the algorithms are tested on the moderate and hard categories, meaning that they still struggle to detect small, occluded and truncated objects. For these reasons, from the authors’ point of view, the current pedestrian and vehicle detection algorithms are still not suitable for AV perception systems. Another reason why the current algorithms are still not suitable for AV perception systems, is that most of the algorithms have been tested in well-recognised datasets such as Caltech and KITTI, and these datasets have their limitations. As observed by Hasan et al. [151], only using these well known datasets to train and test the detection algorithms might limit their generalisation performance.

AVs should meet real-time requirements in order to make quick decisions to avoid collisions. From our review, it is not possible to conclude if the reviewed works meet real-time requirements for AVs, for the following reasons: several works have not reported the hardware they have used and the achieved detection time, for example, Chu et al. [99], Ren et al. [101]; and the hardware reported were not appropriated for AVs since they are large, heavy, not power-efficient, and not cheap. Furthermore, it is not possible to make a fair comparison between the algorithms to decide which one is the fastest, for the reason that the works have used different GPUs and CPUs. For example, Cai et al. [94] reported a detection speed of 0.4 s/img using a NVIDIA Titan GPU, while Liu et al. [102] reported a detection speed of 0.2 s/img using a 1080Ti GPU.

## 7. Conclusions

Pedestrians and vehicles are important objects that AV perception systems must detect. Hence the purpose of this review was to survey the most relevant pedestrian and vehicle detection algorithms. Since several pedestrian and vehicle detection algorithms made use of the same or a modified version of the methods used in generic object detection algorithms, this paper also reviews relevant generic object detection algorithms. The review shows that:

- Nowadays, the preferred methods for object detection are based on DL techniques;
- Even though good results have been achieved for pedestrian and vehicle detection, the current algorithms are still struggling to detect small, occluded and truncated objects;
- There is limited work that investigates how to improve detection performance in bad illumination and weather conditions;
- From the authors’ point of view, the current algorithms are still not ready to be deployed in AV perception systems;
- Using only the traditional datasets (e.g., Caltech, KITTI, etc.) can make the algorithms have limited generalisation;
- It is not possible to make a fair comparison between the achieved detection speed of the algorithms, because they have been trained and tested in different hardware (e.g., GPUs, CPUs, etc.);
- Using techniques, such as ensemble and cascade architectures, to combine different detection algorithms has been shown to improve accuracy performance.

### Suggested Future Research Directions

The authors would recommend the following future works:

- Have more research that adopts ensemble techniques, since there is limited work that did this, and the ones reported in this review achieved improvement on the detection performance;
- Implement pedestrian and vehicle algorithms with more new challenging and large datasets, such as PIE Rasouli et al. [168] and BDD100K Yu et al. [169];
- Adopt cross-dataset evaluation and progressive training pipelines to increase generalisation performance, such as in Hasan et al. [151];
- Explore and improve detection algorithms in scenarios where weather and illumination conditions are challenging;
- Make a comparative study of the reviewed algorithms to report which one gives the best trade-off between accuracy and efficiency. In the comparative study, all the algorithms should be trained and tested on the same set of hardware. Although Haris and Glowacz [23] performed a comparative study of DL algorithms to detect road objects, they only compared the most known general object detection algorithms;
- Analyse if the reviewed algorithms meet AV real-time requirements. The authors would suggest adapting and implementing the reviewed algorithms in embedded devices, such as NVIDIA DRIVE Orin, NVIDIA DRIVE AGX Pegasus, and so forth.

## Figures and Tables

**Table 1:** Benefits and implications of AVs [34,35].

Benefits	Challenges
It is expected that AVs will be able to have vehicle to vehicle/infrastructure (V2V, V2I) communication, therefore, it would be able to choose more efficient routes, reduce or even remove intersection delay and collisions; Make the best use of the road lanes by maintaining short gaps between vehicles; Improve social inclusion since unlicensed, young, disable and elderly people would be able to use them; Improve freight transportation, for example, travel long distance in less time, offer cheaper freight since drivers are not required, and trucks would drive more efficiently; Increase economic opportunities; Reduce parking space; Enhance driver experience by offering comfort during the trip by avoiding harsh braking and jerking; It is estimated that each year driver spend 6 working weeks driving(Perry, 2015). AV could enable people to have free time to relax or work while going to his/her destination; Less $CO_{2}$ emission; AV is expected to reduce traffic accidents hence there will be less expense with legal works and compensations, and car insurance price would be lower.	Currently human drivers are better in recognising pedestrians, cyclists, and other small traffic objects; Human driver is better in recognising different types of materials, for example, if an object ahead is made of cardboard, wood, or concrete. A reasonable quantity of AVs is required to validate their benefits; Not everyone will be able to afford AV technology; Since unlicensed young, elderly, and vulnerable people will be able to travel, this would increase the number of trips and could cause more congestions; Initially public may have some resistance to accept and become comfortable with AVs (Yuen et al., 2020); Initially driver of normal cars would not predict AVs behaviour. Creating new legislation, regulations, certification, testing standards and insurance for AVs; Security against cyber-attacks.

**Table 2:** Advantages and disadvantages of various sensors used in AVs [36].

	Advantages	Disadvantages
CAMERA	-Low cost. -Technology is mature. -High Resolution. -Possible to generate 3D stereoscopic view. -Detect RGB information. -Road markings and signs are design for human eyes. -Less chance to be affected by interference from another vehicles. -Wider field of view.	-Short range. -Performance decreases in poor weather and low light conditions. -Does not provide accurate distance and position of objects. -Usually does not provide depth information. Depth information can be acquired but make the system more complex (e.g., stereo camera and disparity estimation algorithms).
THERMAL	-Distinguish between hot and cold targets. -Can be used during the day and night.	-More expensive than cameras. -Main target is pedestrian but can get confused with hot air from the exhaust pipe or other objects that generates heat. -Cannot detect heat through glass, for example, detect drivers inside the car.
INFRARED	-Can be used during the day and night-time. -They are cheaper and small.	-It has short range.
SONAR	-Cheap.	-Affected by poor weather conditions. -Short range, it is commonly used for automated parking and blind spot detection features.
RADAR	-Its performance is not affected in bad weather or low light conditions. -Long range (up to 300 m). -Provide accurate distance, position, and speed. -Technology is mature. -Low cost	-Limited resolution. -Limited to recognise objects.
LIDAR	-360-degree view of the environment. -Wide field of view. -High accuracy.	-Low spatial resolution compared to cameras. -Performance decreases in poor weather. -Complex and requires high processing power. -Expensive. -Affected by interference and external light. -Does not provide colour information. -Acquired information are sparse.

**Table 3:** Advantages and disadvantages of the different classifiers used in traditional techniques.

Classifier	Advantages	Disadvantages
Linear-SVM	-Use less memory. -Fast to train and classify.	-Lower accuracy.
Non-linear SVM	-Good accuracy.	-Slow to train and test. -Considerable complexity.
Histogram Intersection Kernel SVM (HIKSVM)	-Same computational load as Linear SVM.	-Bad performance when using FPPI evaluation. -It has a short-range.
AdaBoost	-Fast classification.	-Slow to train. -Complexity increases as the number of classes increases. Hence, not suitable for challenging datasets.
MPL-Boost	-Learns Multiple classifiers in parallel.	-Still limited by the number of classes.

**Table 4:** Relevant vehicle detection works using traditional and DL techniques.

	Work	Methods	Algorithm Objective	Dataset-Results
**Traditional Techniques**	VeDAS [85]	**Appearance based** Multi-part based model. Active learning and Symmetry. **Feature extractor**: Haar Like. **Classifier**: AdaBoost. **Hardware**: i7 CPU with 4 cores.	Occlusion handling.	**LISA** True-Positve: 95% Detection-Rate: 87% Time: 15–25 fps.
	[90]	**Appearance based** And-Or model to represent occlusion and context. **Classifier**: Weak-level Structural SVM **Hardware**: NVIDIA GPU. **Evaluation**: AP(%).	Occlusion handling.	**KITTI**: Easy: 84.80% Medium: 75.94% Hard: 60.70% Time: 2 to 3 s/img.
	[83]	**Region proposal**: Edges and Shadows. **Feature extractor**: HOG. **Classifier**: AdaBoost. **Tracking**: Feature points matching using Harris algorithm. **Hardware**:i5 3.2 GHz CPU 4 GB RAM.	Vehicle detection and Tracking for FCW.	**OWN** True Positive: 91.37 False-Positve: 3.09 Recognition Error: under 5%
	[86]	**Feature extractor**: Combination of HOG + LBP + Haar. **Classifier**: AdaBoost. **Hardware**: Intel Xeon 8 core 3.0 GHz.	Comparison of different feature extractors.	**Udacity & KITTI** Precision: 94.7% Detection Rate: 91% Time: 0.528 s/img.
	[84]	**Feature extractor**: HOG (for day-time and dusk). **Classifier**: SVM (for day-time and dusk). **Detector**: DBN (for night-time). **Hardware**: Zynq Soc.	Improve vehicle detection in different light conditions (day, dusk, and dark).	**UPM & SYSU** Accuracy: Day 91.56% Dusk 85.34%. Time: 50 fps/img.
**Deep Learning Techniques**	[92]	**Detector**: 2D DBN. **Hardware**: Advantech industrial computer.	Improve vehicle detection performance by retaining more discriminative information.	**OWN & Caltech** Detection Rate: 96.05% Time: 53 ms/img.
	Faster-RCNN [67]	**Detector**: original Faster R-CNN. **Hardware**: GPU 3.5 GHz. **Evaluation**: AP(%).	Generic object detection.	**KITTI** Easy: 88.97% Medium: 83.16% Hard: 72.62% Time: 2 s/img.
	[93]	**Detector**: Faster R-CNN. **Hardware**: 32 Core server 3.1 GHz, 13 GB RAM, Tesla K40 GPU. **Evaluation**: AP(%).	Handle scale sensitivity. Improve vehicle detection by fine-tuning the Faster R-CNN detector.	**KITTI** Easy: 95.14%. Medium: 83.73%. Hard: 71.22%. Time: 0.32 and 0.47 s/img.
	MS-CNN [94]	**Detector**: MS-CNN. **Feature up-sampling**: Deconvolutional Layer. **Hardware**: Intel Xeon E5-2630 2.40 GHz 64 GB and NVIDIA Titan GPU. **Evaluation**: mAP(%).	Handle scale sensitivity and improve detection speed.	**KITTI** Easy: 90.03%. Medium: 89.02%. Hard: 76.11%. Time: 0.4 s/img.
	DAVE [95]	**Region proposal**: FVPN. **Attributes**: ALN (Based on GoogleNet). **Detector**: Combination of FVP + ALN. **Hardware**: NVIDIA Titan X GPU.	Vehicle detection and pose annotations.	**UTS** AP: 62.85%, 2 fps. **PASCAL** AP: 64.44%, 4 fps. **LISA** AP: 79.41%, 4 fps.
	SDP+CRC(ft) [96]	**Multi-Scale method**: Scale Dependent Pooling (SDP). **Feature Extractor**: VGG-16. **Classifier**: Cascade Rejection Classifier (CRC). **Detector**: SDP-CRC(ft). **Hardware**: NVIDIA K40 GPU. **Evaluation**: AP(%).	Handle scale variance and improve detection speed.	**KITTI** Easy: 90.33% Medium: 83.53% Hard: 71.13% Time: 0.6 s/img.
	[97]	**Region proposal**: Graph based algorithm + Super-pixels. **Detector**: VGG-16. **Hardware**: NVIDIA Titan GPU. **Evaluation**: AP(%).	Decrease the number of Region Proposal candidates.	**KITTI**: Easy: 80.53% Medium: 67.89% Hard: 58.23% Time: 1.57 s/img.
	[98]	**Detector**: R-CNN with modified anchor boxes and use of shallow features. **Hardware**: Not Specified.	Handle multi-scale vehicles.	**KITTI** AP: 83.6% Time: Not Specified.
	RV-CNN. [99]	**Region proposal**: RoI Voting. **Feature extractor**: AlexNet + GoogleNet + Res-50Net. **Detector**: RV-CNN a Multi-task learning and ensemble network. **Evaluation**: AP(%).	Improve vehicle detection robustness.	**KITTI** Easy: 91.28% Medium: 91.67% Hard: 85.43% Time: Not Specified.
**Deep Learning Techniques**	Deep-MANTA [100]	**Object Proposal**: Coarse-to-Fine RPN. **Feature extractor**: GoogleNet or VGG16. **Detector**: Deep Course-to-fine Many task CNN. **Hardware**: Not Specified. **Evaluation**: AP(%).	Improve detection performance and robustness (occlusion and truncation) by performing 2D/3D vehicle analysis.	**KITTI** Easy: 96.40% Medium: 90.10% Hard: 80.79% Time: 0.7–2.1 s/img.
	RRC [101]	**Feature Extractor**: VGGNet. **Detector**: RRC enables feature aggregation to extract more contextual information. Multi-scale feature pyramid. **Hardware**: Not specified. **Evaluation**: AP(%).	Improve mAP for single stage method.	**KITTI** Easy:N\ a Medium: 90.19%. Hard: 86.97%. Time: Not specified.
	ITVD [102]	**Region Proposal**: BFEN. **Feature extractor**: ResNet50. **Detector**:ITVD which is the combination of ResNet-50+ BFEN + STM + SLPN. **Hardware**: GPU 1080Ti. **Evaluation**: AP(%).	Improve detection of small vehicles by acquiring high-quality region proposals.	**KTTI**: Easy: 95.85% Medium: 91.73% Hard: 86.37% Time: 0.3 s/img. **DETRAC**: Easy: 92.95% Medium: 81.43% Hard: 63.73% Time: 0.2 s/img.
	SINet [82]	**Region proposal**: Context-aware pooling to conserve small object properties. **Feature extractor**: VGG or PVA. **Detector**: Multi-branch decision network called SINet. **Hardware**: Intel Xeon E5-1620 3.5 GHz and GPU NVIDIA Titan X. **Evaluation**: AP(%) and mAP(%).	Handle scale sensitivity.	**KITTI**: Easy: 90.60% Medium: 89.60% Hard: 77.75% Time: 0.11–0.2 s/img. **OWN (LSVH)**: mAP: 70.17%.
	AP-SSD [103]	**Feature extractor**: multi-shape and colour Gabor. R-Net to generate AG map. Dynamic Region Enlargement to detect small objects. **Detector**: SSD. **Hardware**: Intel Xeon E5-2603 1.8 GHz and NVIDIA Quadro K620 GPU.	Improve detection accuracy.	**KITTI**: AP: 92.23% Time: 31.86 fps
	Cascade R-CNN [104]	**RoI**: RPN **Detector**: cascades of the Faster R-CNN detector. **Hardware**: Single Titan Xp GPU.	Improve detection quality (increase IoU threshold) by using multistage object detection.	**KITTI** Easy: 90.68%. Medium: 89.95%. Hard: 78.40%. Time: 0.14 s/img.
	[105]	**Feature extractor**: ZFNet **Detector**: Faster R-CNN **Hardware**: Intel Xeon E5-2630 2.40 GHz 64 GR RAM, NVIDIA GTX 1080 GPU.	Vehicle type classification.	**OWN** mAP: 81.05% Time: 0.354 s/img.
**Deep Learning Techniques**	[106]	**Region proposal**: uses a novel part-aware RPN and PAFs to construct feature vectors for different parts of the vehicle. **Feature extractor**: VGG-16. **Detector**: Faster R-CNN. Part-aware NMS to reduce the number of box candidates. **Hardware**: NVIDIA Titan X GPU 12 GB memory. **Evaluation**: mAP(%).	Handle occlusion and truncation.	**KITTI** Easy: 90.21% Medium: 89.01% Hard: 80.72% Time: 2.1 s/img.
	MonoFENet [107]	**Region proposal**: RPN same as Faster R-CNN, RoI max pooling for the feature presentation, and RoI mean pooling for RoI point clouds. **Disparity Estimation**: DORN. **Feature extractor**: VGG-16 or ResNet-101 for image feature, and PointFE Network for point clouds features. **Detector**: MonoFENet. **Hardware**: Not specified. **Evaluation**: AP(%).	Feature enhancement by detecting 3D bounding boxes.	**KITTI** Easy: 91.42% Medium: 84.09% Hard: 75.93% Time: 0.06 s/img.
	SS3D [108]	**Feature extractor**: ResNet34 or drn_c_26 (Dilated Residual network) encoder. **3D boxes Estimator**: non-linear least squares optimizer. **Detector**: SS3D. **Hardware**: Not specified. **Evaluation**: AP(%).	Improve inference time and detection accuracy for monocular 3D vehicle detection.	**KITTI** Easy: 92.72% Medium: 84.92% Hard: 70.35% Time: 0.048 s/img.
	[109]	**Feature extractor**: retrieve-and-transform LTN. **Detector**: Faster R-CNN. **Hardware**: Not specified. **Evaluation**: AP(%).	Trade-off between accuracy and speed.	**KITTI**: Easy: 90.12% Medium: 88.85% Hard: 79.62% Time: 0.4 s/img.
	DLNet [110]	**Detector**: DLNet is a combination of characteristics of DenseNet, YOLO and MobileNet. **Hardware**: GPu GeForce Titan X.	Improve speed and decrease resources requirements.	**CITY** AP: 78%. Time: 71 fps. Weight: 10.1MB.
	[111]	**Feature extractor**: VGG-16. **Detector**: Faster RCNN. **Hardware**: Not specified.	Improve detection accuracy by fine-tuning Faster RCNN.	**CityScapes** AP: 89.06% Time: Not specified.
	[112]	**Feature extractor**: DarkNet53. **Multi-scale method**: Multi-level feature pyramid. **Detector**: YOLOv3. **Hardware**: i7-9700 anbd NVIDIA GTX 1080ti. **Evaluation**: AP(%).	Handle multi-scale vehicles.	**KITTI**: Easy: 95.04% Medium: 92.39% Hard: 87.51% Time: 2.1 s/img.
	[113]	**Feature extractor**: HOG + LBP + Haar Like + VGG. **Detector**: SVM + Cascade CNN. **Hardware**: i7-8700 CPU 3.2 GHz 16 GB memory.	Improve detection accuracy and robustness.	**BDD+Udacity+Other**: Precision: 97.32% Recall: 98.69% Time: Not specified.

**Table 5:** Relevant works for pedestrian detection using traditional techniques.

Work	Feature	Classification	MR
VJ [128]	Appearance + Motion (VJ-rectangles)	Cascade-AdaBoost	94.73%
HOG [47]	HOG	Linear SVM	68.46%
SHAPELET [129]	SHAPELET	AdaBoost	91.37%
LatSvm-V1 [125]	HOG + DPM	Laten SVM	79.78%
HIKSVM [130]	HOG	HIKSVM	73.39%
MULTIFTR [131]	HOG + Shape Context + Haar Features	Linear SVM	68.26%
LatSvm-V2 [126]	HOG + DPM	Latent SVM	63.26%
ChnFtrs [132]	ICF	AdaBoost	56.34%
FPDW [133]	Approximate Multiscale Gradient histograms + ICF	AdaBoost	57.40%
MULTIFTR+MOTION [134]	HOG + Optical Flow + CSS	HIKSVM	50.88%
MLS [135]	Macro-feature (Shapes)	AdaBoost	61.03%
CorssTalk [136]	HOG + LUV + ICF	Crosstalk Cascade	53.88%
SquaresChnFtrs [137]	HOG + LUV	Linear SVM	50.17%
Roerei [137]	HOG + LUV + Multi-Scales + Global Normalisation	Linear SVM	48.50%
ACF [138]	HOG + LUV + Normalised Gradient Magnitude	AdaBoost	51.36%
InformedHaar [139]	Haar-Like + HOG + LUV	AdaBoost	34.60%
LDCF [140]	Same as ACF	Same as ACF	25.0%
Katamari-v1 [6]	HOG + LUV + DCT+ Optical Flow	Not Clear	22.49%
Checkerboards [141]	HOG + LUV (Filtered channels feature)	AdaBoost	18.50%
NNNF-L4 [142]	HOG + LUV NNF: SIDF and SSF	AdaBoost	16.84%
FSSS [143]	ACF + FSSS	RealBoost L3 Decision tree	13.96%

**Table 6:** Relevant works for pedestrian detection using hybrid or DL techniques.

Work	Methods	Algorithm Objective	Dataset & Results
DBN-Isol [127]	**Hybrid** HOG + DPM + Scores of parts + Model-part Visibility Estimation Deep Model Hardware: Not specified. Evaluation: MR(%).	Occlusion handling and deformations.	**Caltech** Reasonable: 61.00% Heavy: 93.00% Time: Not specified.
ConvNet [152]	DL LeNet CNN. Unsupervised Multi-Stage Feature Learning Hardware: Not specified. Evaluation: MR(%).	Improve features.	**Caltech** Reasonable: 77.20% Time: Not specified.
DBN-Multi [153]	**Hybrid** HOG + DPM + Part Detection Score Mutual Visibility Deep model Hardware: 2.27 GHz CPU. Evaluation: MR(%).	Cluttered background.	**Caltech** Reasonable: 48.00% Time: Not specified.
**ContDeepNet or SDP** [154]	**Hybrid** HOG + CSS + Feature Pyramid Deep Model Hardware: Not specified. Evaluation: MR(%).	Improve features.	**Caltech** Reasonable: 45.00% Time: Not specified.
UDN\JointDeep [123]	Hybrid Unify feature extraction, part deformation model, occlusion handling and classification. Standard CNN Back-propagation for Optimisation Hardware: Not specified. Evaluation: MR(%).	Improve features, deformation, occlusion, and classification.	**Caltech** Reasonable: 39.00% Time: Not specified.
SDN [155]	**Hybrid** HOG + CSS + SVM for pruning. Learn Features + Saliency Maps + Mixture Representations Switchable Restricted Boltzmann Machine + CNN Hardware: NVIDIA GTX 760 GPU. Evaluation: MR(%).	Cluttered background and occlusion.	**Caltech** Reasonable: 37.87% Time: <0.1 s/img.
CompACT-Deep [156]	Hybrid ACF + HOG + LUV Complexity aware cascade training (AdaBoost) Hardware: Intel Xeon E5-2620 64 GB RAM and NVIDIA Tesla K40m GPU. Evaluation: AP(%) for KITTI and MR(%) for Caltech.	Trade-off between accuracy and complexity.	**Caltech** Reasonable: 11.7% **KITTI** Easy: 70.69% Medium: 58.74% Hard: 52.71% Time: 1 s/img.
MS-CNN [94]	**DL** Multi-Scale (Uses earlier layers of the Network since it is better to detect small objects) Proposal and Detection network. Deconvolutional Layer to increase feature map resolution. Hardware: Intel Xeon E5-2630 64 GB RAM and NVIDIA Titan GPU. Evaluation: AP(%) for KITTI and MR(%) for Caltech.	Variance in instance scale and fast detection.	**Caltech** Reasonable: 9.95% **KITTI** Easy: 83.92% Medium: 73.70% Hard: 68.31% Time: 0.4 s/img.
RPN+BF [157]	**DL**. RPN region proposal BF to mine hard negative examples. Faster-RCNN for detection. Hardware: Tesla K40 GPU. Evaluation: AP(%) for KITTI and MR(%) for Caltech.	Variance in instance scale.	**Caltech** Reasonable: 9.60% Time: 0.5 s/img. **KITTI** Easy: 77.12% Medium: 61.15% Hard: 55.12% Time: 0.6 s/img.
MCF [158]	Hybrid Multi-layer Channel features Integrates HOG+LUV channels + the CNN layers Multistage cascade AdaBoost as the learn and classification algorithm. Hardware: Intel Core i7-3700. Evaluation: MR(%).	Improve features.	**Caltech** Reasonable: 10.40% Time: 0.54 fps.
F-DNN and F-DNN+SS [148]	DL -Ensemble. SSD for pedestrian candidate generator. Multiple binary classification DNNs (Resnet and GoogLeNet). SNF for network fusion. Semantic Segmentation to refine object detection. Hardware: NVIDIA Titan X GPU. Evaluation: MR(%).	Fast and robust.	**Caltech** F-DNN: 8.65% F-DNN+SS: 8.18% Time: 2.48 s/img.
SA Fast-RCNN [122]	Hybrid ACF for region proposals. Combine a large-size and a small-size sub-network. Divide-and-conquer philosophy. Hardware: NVIDIA GeForce GTX Titan X GPU 12 GB. Evaluation: AP(%) for KITTI and MR(%) for Caltech.	Variance in instance scale.	**Caltech** Reasonable: 9.32% **KITTI** Easy: 77.93% Medium: 65.01% Hard: 60.42% Time: 0.59 s/img.
SDS-RCNN [150]	DL -Ensemble Semantic Segmentation. RPN+BF region proposal. Hardware: Titan X GPU. Evaluation: AP(%) for KITTI and MR(%) for Caltech.	Detection accuracy.	**Caltech** Reasonable: 7.36% **KITTI** Medium: 63.05% Time: 0.21 s/img.
ADM [146]	**DL -RNN** Resnet to extract features R-CNN for pedestrian proposals. Active detector using RNN (LSTM) to improve bbox predictions. Hardware: Not specified. Evaluation: MR(%).	Variance in instance scale.	**Caltech** Reasonable: 9.0% Time: Not specified.
PCN [147]	DL -RNN Part and Context Network. RNN (LSTM) for semantic information. Hardware: Not specified. Evaluation: MR(%).	Occlusion handling.	**Caltech** Reasonable: 8.4% Time: Not specified.
GDFL [159]	**DL** VGG16 to extract feature. Scale-aware pedestrian attention module. Zoom-in-Zoom-out module using max-pooling and bi-linear interpolation. Hardware: Single GeForce GTX 1080Ti GPU. Evaluation: AP(%) for KITTI and MR(%) for Caltech.	Improve feature extraction.	**Caltech** Reasonable: 7.84% **KITTI** Easy: 84.61% Medium: 68.62% Hard: 66.86% Time: 20 fps.
F-DNN2 SS [149]	DL -Ensemble SSD to generate pedestrian candidates. Soft-rejection to adjust confidence. Semantic Segmentation. Hardware: Single NVIDIA Titan X GPU. Evaluation: AP(%) for KITTI and MR(%) for Caltech.	Detection accuracy and velocity.	**Caltech** Reasonable: 7.67% **KITTI** Easy: 74.05% Medium: 61.17% Hard: 57.15% Time: 2.48 s/img.
TLL-TFA [160]	Topological annotation to introduce less ambiguity. MRF to eliminate ambiguities in occlusion cases. Temporal feature aggregation as an extra feature. Hardware: Not specified. Evaluation: MR(%).	Variance in instance scale.	**Caltech** Reasonable: 7.40% Time: Not specified.
AR-Ped [145]	DL -Ensemble + Encoder-Decoder + Cascade + RNN Auto-regressive RPN. Decoder-encoder module for feature refinement. R-CNN classifier. Hardware: NVIDIA 1080Ti GPU. Evaluation: AP(%) for KITTI and MR(%) for Caltech.	Improve features.	**Caltech** Reasonable: 6.45% **KITTI** Easy: 83.66% Medium: 73.44% Hard: 68.18% Time: 91ms/img.
Parallel-Net [161]	DL Fire modules to reduce parameters. Feature Fusion: Parallel convolutional layers are combined. Detector: Parallel-Net. Hardware: NVIDIA 2080Ti GPU. Evaluation: AP(%) for KITTI and MR(%) for Caltech.	Improve features and reduce parameters.	**Caltech** Reasonable: 54.61% Time: 35.71 fps. **KITTI** Combined: 77.90%. Time:2.26 fps.
Adaptive Perceive -SSD [103]	See Table 4 Evaluation: AP(%).	See Table 4	**KITTI** Combined: 92.42%.
CSP Liu et al. [162]	Detector:Center Scale Prediction (CSP). Resnet to extract centre points that can represent heat and scale map of the pedestrians. Hardware: GTX 1080Ti GPU. Evaluation: MR(%).	Avoid the use of sliding windows or anchor boxes.	**Caltech** Reasonable: 4.5% Time: 0.33 s/img.
W${}^{3}$-Net Luo et al. [163]	Feature Extractor: ResNet-50 + FPN. Detector: Where, what, and Whether Network (W${}^{3}$-Net). GAN transforms 2D images to Bird-view-map. Transform from bird view to front view map to create a relationship between object scale and depth. Encoder-Decoder technique to re-encode body parts into full body. Hardware: NVIDIA GTX 1080Ti. Evaluation: MR(%).	Handling occlusion.	**Caltech** Reasonable: 6.37% Heavy: 28.33% **CityPerson** Reasonable: 9.3% Heavy: 18.7% Time: 0.31 s/img.
TFAN Wu et al. [164]	Feature Extractor: ResNet. Detector:Tube feature aggregation network (TFAN). Temporal feature fusion: temporally discriminative embedding module and part-based relation module. Hardware: Not specified. Evaluation: MR(%).	Make use of temporal context information to handle heavy occlusion.	**Caltech** Reasonable: 6.5% Heavy: 31.5% Time: Not specified.
MFPN [165]	Resnet to extract features. Feature Fusion: Bi-direction FPN to acquire more semantic information. Repulsion Loss of Minimum to increase detection quality. Hardware: i7-6500 CPU 2.5 GHz and NVIDIA GTX 1080Ti GPU. Evaluation: AP(%).	Handling occlusion.	**CrowdHuman** 90.96%. Time: Not specified.
DAGN and DAGN++ Xie et al. [166]	Detector: cascade R-CNN. Tone mapping to deal with poor illumination. Deformable Convolution with an Attention Module. DAGN++ uses progressive training pipeline. Hardware(Testing): Single GTX Titan X GPU. Evaluation: MR(%).	Handling occlusion.	**Caltech** DAGN: 6.03% DAGN++: 1.84% Time: 0.11 s/img.
Pedestron Hasan et al. [151]	Cross-dataset evaluation. Progressive training pipeline. Detector: cascade R-CNN. Hardware: Not specified. Evaluation: MR(%).	Handling generalisation limitations.	**Caltech:** Reasonable: 2.5% Small: 9.9% Heavy: 31.0% **Cityperson:** Reasonable: 9.7% Small: 11.8% Heavy: 31.0% Time: Not specified.

## Data Availability

Not applicable.

## Abbreviations

The following abbreviations are used in this manuscript:

AV	Autonomous Vehicle.
DL	Deep Learning.
KITTI	Karlsruhe Institute of Technology and Toyota Technological Institute
PIE	Pedestrian Intention Estimation.
BDD100K	Berkeley DeepDrive.
ADAS	Advanced Driver Assistance System.
HDAI	Hazard Detection System.
DARPA	Defence Advanced Research Projects Agency.
SAE	Society of Automotive Engineers.
V2V	Vehicle to Vehicle.
V2I	Vehicle to Infrastructure.
SLAM	Simultaneous Localisation and Mapping.
CDD	Charge-coupled Devices.
CMOS	Complementary Metal-oxide Semiconductors.
LIDAR	Light Detection and Ranging.
RADAR	Radio Detection and Ranging.
IMU	Inertial Measurement System.
GNSS	Global Navigation Satellite Systems.
ML	Machine Learning.
CNN	Convolutional Neural Network.
PID	Proportional Integral Derivative.
SIFT	Scale Invariant Feature Transform.
HOG	Histogram of Oriented Gradient.
EOH	Edge-Orientation-Histograms.
ISM	Implicit Shape Model.
SSC	Self-similarity Channels.
SURF	Speeded Up Robust Features.
MSER	Maximally Stable Extremal Regions.
ICF	Integral Channels Features.
ACF	Aggregated Channel Features.
SVM	Support-Vector-Machine.
AdaBoost	Adaptive Boosting.
ANN	Artificial Neural Network.
BBF	Best-Bin-First.
HT	Hough Transform.
ILSVRC	ImageNet Large-Scale Visual Recognition Challenge.
R-CNN	Region Convolutional Neural Network.
SPP-Net	Spatial Pyramid Pooling Network.
RoI	Region of Interest.
RPN	Region Proposal Network.
FPS	Frame Per Second.
FPN	Feature Pyramid Network.
YOLO	You Look Only Once.
SSD	Single Shot Multi-box Detection.
LIEM	Local Information Extraction Model
GIEM	Global Information Extraction Model
DNN	Deep Neural Network.
MSF	Multi-scale Features.
EMA	Exponential Moving Average.
IoU	Intersection Over Union.
ITS	Intelligent Transportation Systems.
LBP	Local Binary Patterns.
PCA	Principal Component Analysis.
2D-DBN	2D Deep Belief Network.
BFEN	Backward Feature Enhancement Network.
SLPN	Spatial Layout Preserving Network.
FC	Fully Connected Layer.
RRC	Recurrent Rolling Convolution.
mAP	mean Average Precision.
MS-CNN	Multi-scale CNN.
SDP + CRC	Scale Dependent Pooling and Cascade Rejection Classifiers.
DeepMANTA	Deep Many-Tasks.
monoFET	monocular Feature Enhancement Networks
DORN	Deep Ordinal Regression Network.
SS3D	Single Stage Monocular 3D.
TP	True Positive.
DR	Detection Rate.
AP	Average Precision.
FP	False Positive.
RE	Recognition Error.
ITVD	Imporving Tiny Vehicle Detection.
PAFs	Part Affinity Fields.
DPM	Deformable Part Model.
MR	Miss Rate.
RNN	Recurrent Neural Network.
LSTM	Long Short Term Memory.
SINet	Scale-insensitive Convolutional Neural Network.
LSVH	Large Scale Variance Highway.
RV-CNN	Region of Interest Voting CNN.
UTS	Urban Traffic Surveillance.
FVPN	Fast Vehicle Proposal Network.
ALN	Attributes Learning Network.
DCT	Discrete Cosine Transform.
CSS	Self-similarity on colour channels.
NNF	Non-Neighbouring Features.
SIDF	Sine-Inner Difference Features.
SSF	Symmetrical Similarity Features.
FSSS	Feature Selected Self-Similarity.
SDN	Switchable Deep Network.
BF	Boosted Forest.
SNF	Soft-rejection Network Fusion.
SDS	Simultaneous Detection and Segmentation.
MRF	Markov Random Field.
AG	Accuracy Gain.
